# Effects of experiencing the COVID-19 pandemic on optimistically biased belief updating

**DOI:** 10.7554/eLife.101157

**Published:** 2025-06-30

**Authors:** Iraj Khalid, Orphee Morlaas, Hugo Bottemanne, Lisa Thonon, Thomas Da Costa, Philippe Fossati, Liane Schmidt

**Affiliations:** 1 Control-interception-attention team, Paris Brain Institute (ICM), UMR 7225, U1127, Institut National de la Santé et de la Recherche Médicale/Centre National de la Recherche Scientifique/Sorbonne Universités, Hôpital Pitié-Salpêtrière Paris France; 2 Département de Psychiatrie Adulte, Hôpital Pitié-Salpêtrière, Assistance Publique Hôpitaux de Paris (APHP) Paris France; Stem-cell and Brain Institute (SBRI), U1208 Inserm France; State University of New York Upstate Medical University United States

**Keywords:** belief-updating, Covid-19, optimism bias, reinforcement-learning, Bayesian modeling, computational modeling, Human

## Abstract

Optimistically biased belief updating is essential for mental health and resilience in adversity. Here, we asked how experiencing the COVID-19 pandemic affected optimism biases in updating beliefs about the future. One hundred and twenty-three participants estimated the risk of experiencing adverse future life events in the face of belief-disconfirming evidence either outside the pandemic (n=58) or during the pandemic (n=65). While belief updating was optimistically biased and Reinforcement-learning-like outside the pandemic, the bias faded, and belief updating became more rational Bayesian-like during the pandemic. This malleability of anticipating the future during the COVID-19 pandemic was further underpinned by a lower integration of positive belief-disconfirming information, fewer but stronger negative estimations, and more confidence in base rates. The findings offer a window into the putative cognitive mechanisms of belief updating during the COVID-19 pandemic, driven more by quantifying the uncertainty of the future than by the motivational salience of optimistic outlooks.

## eLife digest

Anticipating the future is an essential part of human thinking and helps us guide how we understand the world. We continuously update this information to make better predictions and generalizations.

When faced with new information, we tend to downplay bad news and emphasize good news. This bias towards optimism supports mental health and helps people stay motivated and resilient when facing difficulties.

However, in hard times, and/or when people feel threatened or depressed, they may lose their ability to focus on good news. Instead, they may begin to emphasize possible negative outcomes. For example, the COVID-19 pandemic created a long-lasting period of challenges for many people. Every-day activities, like going to work or food shopping, became risky. Uncertainty about the economy and health impacts further weighed on people’s minds.

Khalid et al. set out to understand if extreme and long-lasting challenges affect the optimism bias in belief updating. The experiments compared how 93 adults estimated their chances of adverse experiences like a job loss or illness during the pandemic in France, with the estimations of 60 individuals before or after the pandemic. More specifically, 30 people were recruited in France in October 2019 before the pandemic, and a further 34 and 31, respectively, were recruited in March to April 2020 and May 2021, and in addition 28 people from the pre-pandemic group were consulted again in March to April 2020. Another 30 people were recruited after the pandemic in June 2022.

The experiments measured how much people’s predictions changed after learning real statistics about the likelihood of adverse future life events. Before and after the pandemic, people showed a strong optimistic bias when considering these statistics. They gave more weight to good news than to bad news. But this bias disappeared during the pandemic, and people weighed positive and negative information more evenly.

The experiments also showed that before and after the pandemic, people updated their beliefs in a way that resembled trial-and-error learning. In contrast, during the pandemic, their updates were more cautious and evidence-based, like weighing all the facts before making a judgment.

Khalid et al. shed light on how a large-scale crisis can affect our perception of the future. In the long term, this could help mental health professionals better understand how people cope with uncertainty and guide efforts to strengthen resilience in difficult times. More research is needed to explore how changes in belief updating relate to mental health and what it means to adopt a healthy level of optimism to recover after significant life challenges.

## Introduction

Anticipating the future is an essential part of human thinking. These beliefs guide how we understand the world; updating them is vital for learning to make better predictions and to generalize across contexts. Interestingly, belief updating tends to be optimistically biased (Weinstein, 1980). Even when confronted with negative information, humans often downplay its importance to maintain an optimistic view of the future. The underlying mechanism of optimistically biased belief updating involves an asymmetry in learning from positive and negative belief-disconfirming information (Sharot et al., 2011; Sharot and Garrett, 2016; Kuzmanovic et al., 2018), which can unfold in two ways, following Reinforcement learning (RL) or Bayes rule (Kuzmanovic and Rigoux, 2017).

Conceptually, Reinforcement learning (RL) and Bayesian models of belief updating are complementary but make different assumptions about the hidden process humans may use to adjust their beliefs when faced with information that contradicts them. The RL models assume that belief updating is proportional to the estimation error. The key idea of the estimation error expresses the difference between how much someone believes, for example, they will experience a future life event and the actual prevalence of the event in the general population. This difference can be positive or negative. A scaling and an asymmetry parameter quantify the propensity to consider the estimation error magnitude and its valence, respectively. These two free parameters form the learning rate, which indicates how fast and biased participants update their beliefs.

In contrast, Bayesian models assume that following Bayes’ rule the posterior, updated belief is a new hypothesis, formed by pondering prior knowledge with new evidence. The prior knowledge consists in information about the prevalence of life events in the general population. The new evidence comprises various alternative hypotheses. It examines how likely a specific event is to occur or not occur for oneself, compared to the likelihood that it will happen or not happen to others. This probabilistic adjustment of beliefs about future life events can be considered as an approximation of a participant’s confidence in the future. The two free parameters of the Bayesian belief updating model scale how much the initial belief deviates from the updated, posterior belief (i.e. scaling parameter) and the propensity to consider the valence of this deviance (i.e. asymmetry parameter).

Although RL-like and Bayesian updating models make different assumptions about the updating strategy, they are complementary and powerful formalizations of human reasoning. Both models provide insight into hidden, latent variables of the updating process. Most notably, the learning rate and its components, the scaling and asymmetry parameters, can vary between individuals and contexts and, through this variance, offer possible explanations for the idiosyncrasy in belief-updating behavior and its cognitive biases.

The COVID-19 pandemic represented a chronic adverse life context, drastically altering individual and social realities. The existing literature documenting the impact of the COVID-19 pandemic and the associated changes in everyday life on mental health has shown that stress, anxiety, and depression have increased during the pandemic (Rajkumar, 2020; World Health Organization, 2022). Moreover, previous work has demonstrated that belief updating during reversal learning became more erratic and was linked to a rise in paranoia during the COVID-19 pandemic across the US (Suthaharan et al., 2021).

However, it is unknown how optimism biases in belief updating about the future and their underlying putative mechanisms changed during the experience of such an unprecedented, adverse life event. Our hypothesis was twofold. We argued that maintaining optimistically biased belief updating under lasting, adverse life conditions is adaptive. The optimism biases are beneficial in exploratory behavior, reduce stress, and improve mental and physical health and well-being (Taylor and Brown, 1988; Scheier et al., 1994; Scheier et al., 2001; Boehm and Lyubomirsky, 2008; Carver et al., 2010; Carver and Scheier, 2014; Boehm et al., 2018). These benefits promote resilience, which is especially important for fitness and survival during a pandemic (Berger-Tal and Avgar, 2012; McKay and Dennett, 2009). On the contrary, optimism biases can lead to suboptimal decision-making. Contextual factors such as acute stress, perceived threat, and depression have been shown to reduce or even reverse optimistically biased belief updating (Strunk et al., 2006; Garrett et al., 2014; Korn et al., 2014; Garrett et al., 2018; Kuper-Smith et al., 2020; Czekalla et al., 2021; Bottemanne et al., 2022). These findings suggest that optimistically biased belief updating should be weaker when experiencing a pandemic.

We leveraged a belief-updating dataset from 123 participants tested between 2019 and 2022 to rule between these alternative hypotheses. Among them, 58 participants were tested outside the context of the COVID-19 pandemic, either in October 2019, 3 months before the outbreak in France (n=30) or 2 years after in June 2022 (n=28), after the lift of the sanitary state of emergency. Their belief updating behavior was compared to 65 participants tested during the sanitary state of emergency due to the COVID-19 outbreak in France. This was either during the first very strict lockdown of social and economic life (e.g. schools closed, stay-at-home orders, shops, and museums closed) from March to April 2020 (n=34) or 1 year later in May 2021 (n=31), when lockdown was less strict (e.g. schools open, museums and shops closed, part-time curfew), but the COVID-19 pandemic was still unfolding. Belief updating was measured by a behavioral task that asked participants to estimate their risk of experiencing adverse future life events before and after receiving information about these events' actual base rates. Observed belief updating behavior was fitted to an RL-like and a Bayesian updating model to gain insight into potential underlying strategies of belief updating. The learning rates were compared across groups for insight into how experiencing the COVID-19 pandemic changed beliefs about the future and their updating in the face of belief-disconfirming evidence.

## Results

### Effects of experiencing the COVID-19 pandemic on optimistically biased belief updating

**Figure 1.**
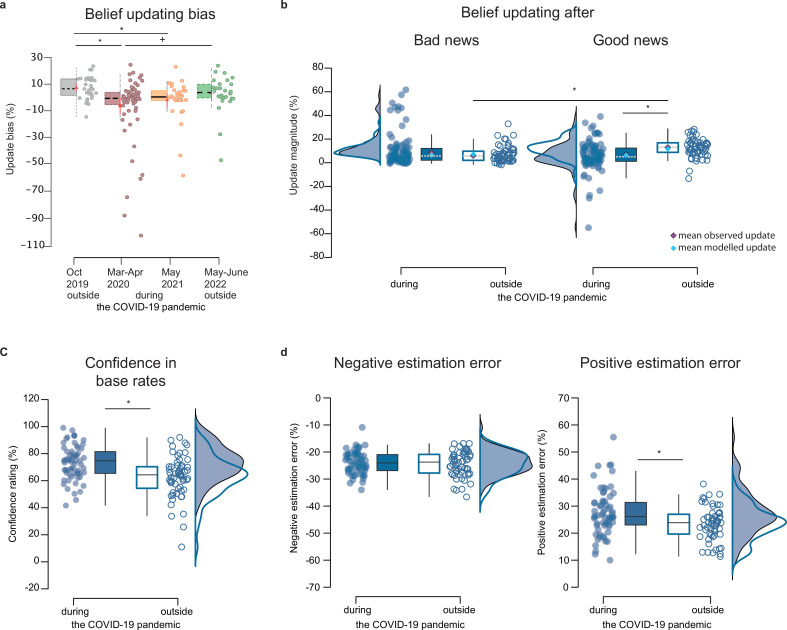
Behavioral results. (**a**) *Boxplots display the belief-updating bias* (i.e. the difference between the belief update for good news and belief update for bad news) in each of the four participant groups, tested before the pandemic in October 2019 (n=30), during the first lockdown from March to April 2020 (n=34), with less restrictive measures in May 2021 (n=31), and at the end of the pandemic in June 2022 (n=28). (**b**) *Belief updating for good and bad news* during (n=65) and outside the pandemic (n=58). (**c**) *Confidence ratings*, and (**d**) *estimation errors for bad and good news during and outside the pandemic*. Boxplots in all panels display 95% confidence intervals, with boxes indicating the interquartile range from Q1 25^th^ to Q3 75^th^ percentile. The horizontal black lines indicate medians, and whiskers range from minimum to maximum values and span 1.5 times the interquartile range. The dots correspond to individual participants. The squares in the boxplots in (**b**) correspond to mean observed updates (purple) and mean modelled updates (blue; averaged across 1000 estimations) from the best-fitting models in each context, which were the optimistically biased RL-like model of belief updating outside and the rational Bayesian model of belief updating during the Covid-19 pandemic. The source data file provides exact values. *<0.05 two-sampled, two-tailed t-tests, * p<0.05 two-sampled, one-tailed t-tests. Figure 1—source data 1.Average values across trials for each participant on behavioral outcome measures.

A linear mixed effects (LME) model was fitted to belief updates to test whether belief updating was less or more biased during the COVID-19 pandemic. The model found a significant interaction estimation error valence by context (ß=–5.54, SE = 1.69, t(232) = –3.28, p=0.001, 95% CI [-8.87 to –2.21]; Appendix 7—table 2), which holds when further controlling for the distance variable (Appendix 7—table 3). The power of this effect was 75% leaving a 25% risk for type II errors. As shown in Figure 1a and b, optimistically biased belief updating disappeared during the COVID-19 pandemic compared to participants tested outside the pandemic. More specifically, it was decreased among participants tested during the initial COVID-19-related strict lockdown in March and April 2020 (EE valence by context 1: ß=–7.39, SE = 2.29, t(228) = –3.21, p=0.002, 95% CI [-11.91 to –2.86]; Appendix 7—table 4), as well as in May 2021 (EE valence by context 2: ß=–5.59, SE = 2.36, t(228) = –2.37, p=0.02, 95% CI [-10.24 to –0.93]; Appendix 7—table 4), compared to those tested before the outbreak in October 2019, respectively. The bias re-emerged among participants tested one year later at the time of the lift of the sanitary state of emergency in June 2022, returning to levels akin to those observed before the pandemic in October 2019 (EE valence by context 3: ß=–2.11, SE = 2.46, t(228) = –0.86, p=0.39, 95% CI [-6.95 to 2.73]; Figure 1a, Appendix 7—table 4). The effect of the COVID-19 pandemic on belief updating was driven by a significant decrease in belief updating following good news during the pandemic compared to participants tested outside the pandemic (t(121) = 2.66, p=0.009, Cohen’s d=0.48, two-sampled, two-tailed t-test, Figure 1b). No contextual group difference was observed for belief updating following bad news (t(121) = –1.77, p=0.08, Cohen’s d=–0.32, two-sampled, two-tailed t-test, Figure 1b). This effect could be reproduced when fitting an analogous LME to belief updates observed in the group of participants (n=28) who were tested both before and during the pandemic (EE valence by context interaction: ß=–7.66, SE = 1.49, t(103) = –5.13, p=1.35e-06, 95% CI [-10.62 to -4.70]; Figure 1—figure supplement 1, Appendix 7—table 5, Appendix 1). Moreover, previous studies of optimistically biased belief updating calculated the estimation error (EE) on the difference between the estimate for someone else (eBR) and the base rate (BR), following: EE = eBR - BR (Kuzmanovic et al., 2018; Kuzmanovic and Rigoux, 2017; Garrett and Sharot, 2014; Kuzmanovic et al., 2015). When categorizing trials as good or bad news based on this alternative EE calculation, the context-by-EE valence interaction remained significant (Appendix 7—table 6). Note that all effects were controlled for participants' age, years of higher education, gender, confidence in the base rates, belief updating task design, and estimation error magnitude.

**Figure 1—figure supplement 1.**
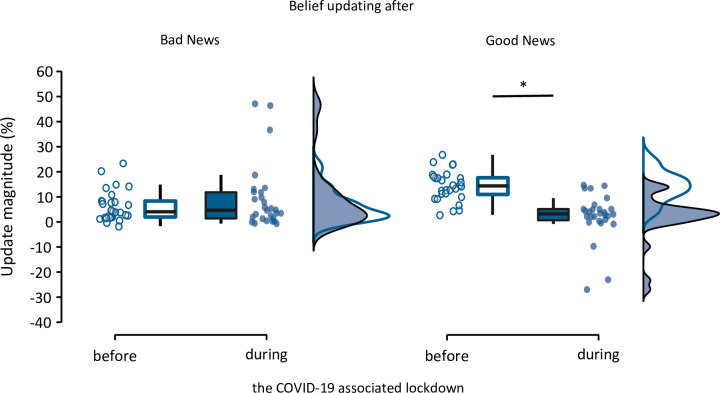
Belief updating within the same group of participants tested before and during the COVID-19 pandemic (n=28). Boxplots display 95% confidence intervals for belief updating after bad (left panel) and good (right panel) news and during and outside the pandemic. Boxes indicate the interquartile range from Q1 25th to Q3 75th percentile. The horizontal black lines indicate medians and whiskers range from minimum to maximum values and span 1.5 times the interquartile range. The dots correspond to individual participants. The source data file provides exact p-values. *p < 0.05 two-sampled, two-tailed t-tests. Figure 1—figure supplement 1—source data 1.Average belief update across trials for each participant tested both before and during the pandemic (within-subjects).

**Figure 1—figure supplement 2.**
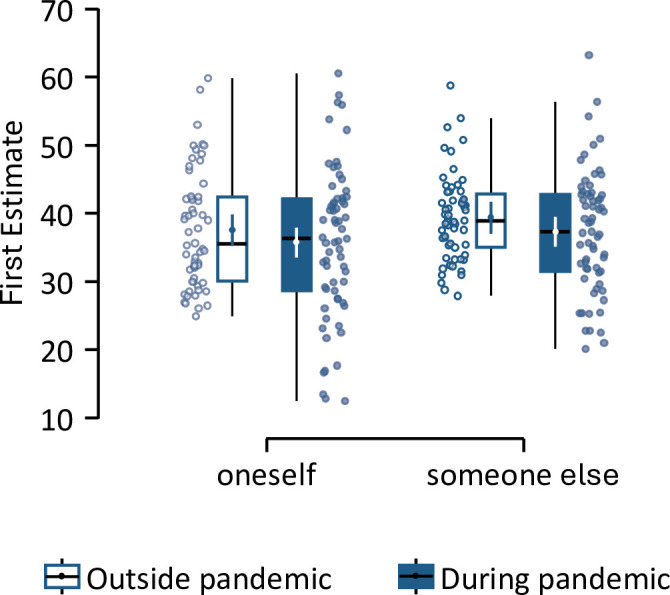
Optimism bias in initial beliefs about adverse future life events. First estimates of the likelihood of and adverse life event happening to oneself (left) or someone else (right) and before (n=58) and during (n=65) the COVID-19 pandemic. Boxplots display 95% confidence intervals with boxes indicating the interquartile range from Q1 25th to Q3 75th percentile. The horizontal black lines indicate medians and whiskers range from minimum to maximum values and span 1.5 times the interquartile range. The individual dot and vertical line in the middle correspond to the means and standard errors. The contiguous dots correspond to individual participants. Figure 1—figure supplement 2—source data 1.Average first estimate across trials for each participant.

### Effects of experiencing the COVID-19 pandemic on belief updating variables

As shown in Figure 1c, experiencing the COVID-19 pandemic influenced participants' confidence in the base rates, with significantly lower confidence ratings observed among those tested outside the pandemic compared to those tested during it (ß=14.11, SE = 4.52, t(233) = 3.12, p=0.002, 95% CI [5.19 to 23.02]; Appendix 7—table 7). Moreover, a significant interaction of EE valence by context (ß=2.19, SE = 0.67, t(233) = 3.28, p=0.001, 95% CI [0.88 to 3.51]; Appendix 7—table 8) was found for absolute estimation error magnitude. This finding indicated that participants tested during the pandemic overestimated their risk of experiencing adverse future life events relative to base rates more largely than participants tested outside the pandemic (t(121) = –3.01, p=0.003, Cohen’s d=–0.54, Figure 1d). On the contrary, the two groups did not differ significantly in the magnitude of negative estimation errors (i.e. initial underestimations relative to base rates; t(121) = –0.49, p=0.63, Cohen’s d=–0.09, two-sampled, two-tailed t-tests; Figure 1d). This finding contrasts with the observed difference in how often they made positive estimation errors (i.e. the number of good news trials). Participants tested during the pandemic overestimated less frequently than participants tested outside the pandemic (t(121) = 2.40, p=0.02, Cohen’s d=0.43, two-sampled, two-tailed t-test). No significant difference between groups was found for the frequency of underestimations (i.e. reflected by the number of bad news trials; t(121) = –1.85, p=0.07, Cohen’s d=–0.33, two-sampled, two-tailed t-test). These results indicated that participants held fewer but stronger negative future outlooks during the pandemic compared to those tested outside the pandemic.

### Effects of experiencing the COVID-19 pandemic on putative mechanisms of belief updating

**Figure 2.**
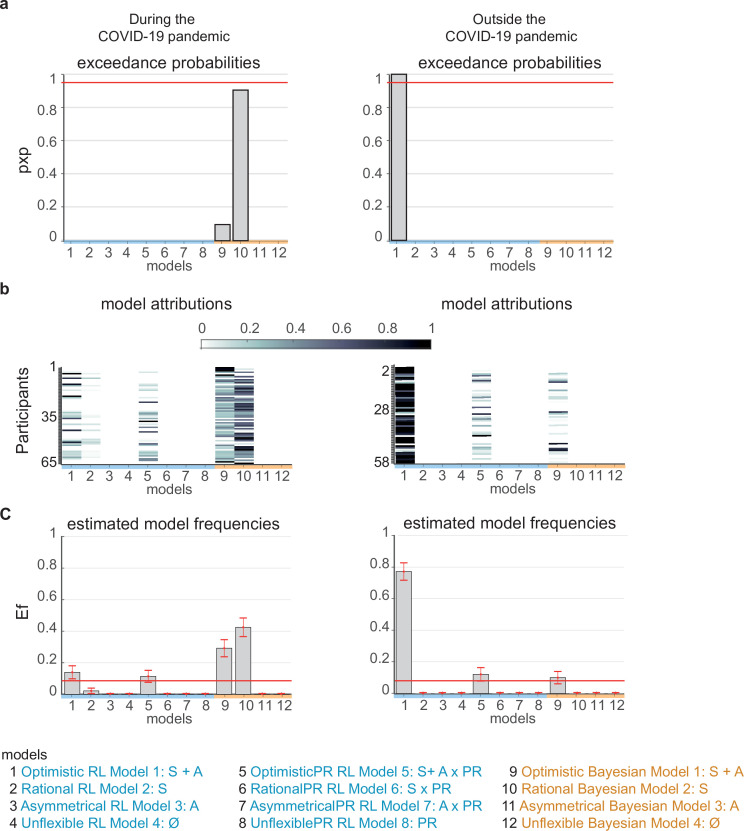
Computational model comparisons. Twelve alternative models from RL-like (blue) and Bayesian (orange) updating model families were fitted to observed belief updates for participants tested during the COVID-19 pandemic (left panel columns) and outside the pandemic (right column panels). (**a**) *Protected exceedance probabilities for each of the 12 alternative models*, which is the probability that the model predominates in the population above and beyond chance. (**b**) *Posterior model attributions*. Colored cells display the probability that individual participants (y-axis) will be best explained by a model version (x-axis). (**c**) *Estimated model frequencies* correspond to how many participants are expected to be best described by a model version, with error bars corresponding to standard deviations. The red line indicates the null hypothesis that all model versions are equally likely in the cohort (chance level). Labels on the x-axis of the histogram and bar graphs indicate the model versions with non-silenced parameters (S – scaling, A – asymmetry) and PR – personal relevance of events. The source data file provides exact values. Figure 2—source data 1.Model comparison metrics.

We then sought to identify which putative strategy participants used to update their beliefs about the future during and outside the pandemic. To answer this question, we used computational modeling and model comparisons to rule between 12 alternative models. This approach revealed that belief updating outside the pandemic was more RL-like and optimistic (pxp = 1, Ef = 0.77), while during the pandemic, it was best explained by a rational Bayesian updating model (pxp = 0.90, Ef = 0.43; Figure 2a–c). Similar findings were obtained when conducting model comparisons in the participants tested both before and during the lockdown (n=28; Appendix 1 , Figure 2—figure supplement 1).

**Figure 2—figure supplement 1.**
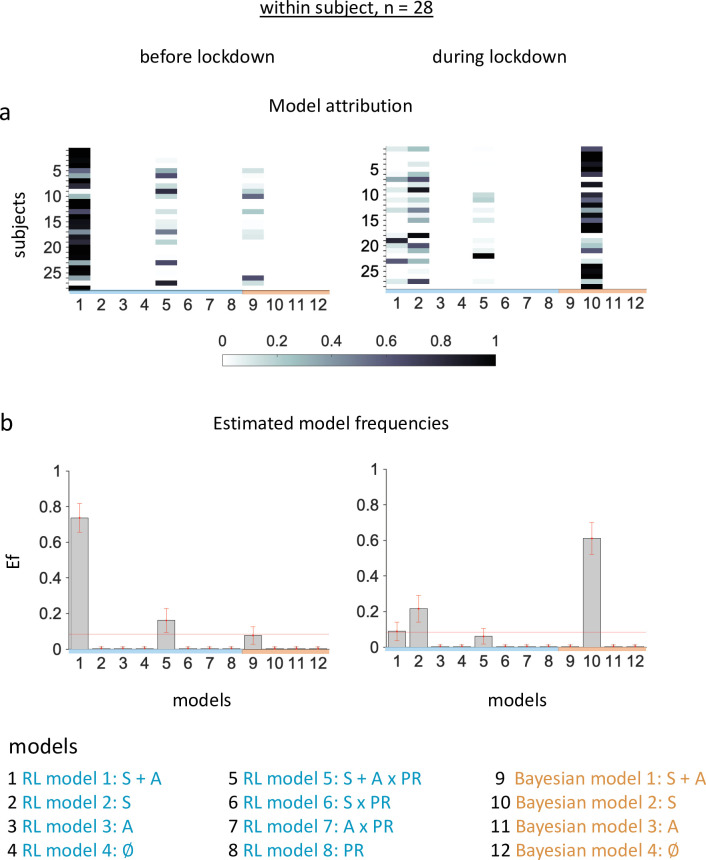
Estimated model frequencies for participants tested both before and during the COVID-19 pandemic. (**a**) Posterior model attributions. Colored cells display the probability that individual participants (y-axis) will be best explained by a model version (x-axis). (**b**) Estimated model frequencies. The histograms display average posterior model frequencies that reflect how many participants are expected to be best described by a model version, with error bars corresponding to standard deviations. The red line indicates the null hypothesis that all model versions are equally likely in the cohort (chance level). Labels on the x-axis of the histograms indicate the model versions with non-silenced parameters (S – scaling, A–asymmetry), and PR – personal relevance factor. Figure 2—figure supplement 1—source data 1.Model comparison metrics for the within-subjects analysis.

**Figure 2—figure supplement 2.**
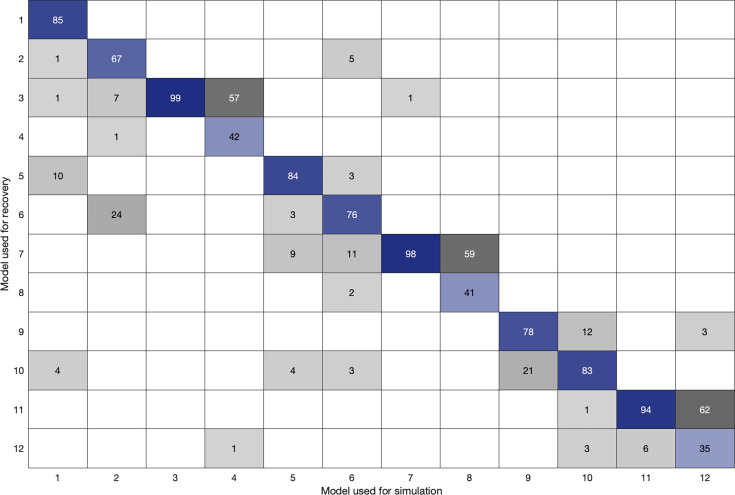
Model recovery confusion matrix. The matrix displays the estimated model frequencies from the model recovery analysis. Each column represents the generative model used to simulate behavioral data, while each row indicates the model used to recover data during the fitting procedure. Higher values along the diagonal (blue) indicate successful recovery, confirming that each model can be reliably distinguished from the others. Off-diagonal values (gray) reflect potential misattributions. Figure 2—figure supplement 2—source data 1.Estimated model frequencies from the model recovery analysis.

**Figure 2—figure supplement 3.**
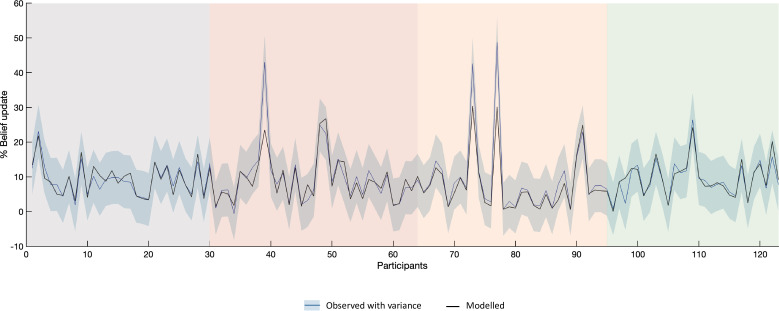
Observed and modelled belief updating for the whole participant sample (n=123). This figure illustrates the percentage of belief update for each participant (blue line) and the estimated belief update (black line) from the overall best fitting optimistically biased RL-like model of belief updating. The shaded blue area reflects the variance in observed data. The colored background highlights the four groups of participants tested in different contexts – before the COVID-19 pandemic (gray), during the 1st lockdown (red), at time of last lockdown release (beige), and one year later (green). Figure 2—figure supplement 3—source data 1.Average observed and modelled belief update across trials for each participant.

### Effects of experiencing the COVID-19 pandemic on hidden, latent variables of belief updating

Next, we compared the effects of experiencing the COVID-19 pandemic on the learning rates and its components. To show that this adverse context effect was indeed mediated by alterations in asymmetrical learning, we compared the scaling and asymmetry parameters obtained from the overall best-fitting model across the whole dataset of n=123 participants. This was Model 1 – the optimistically biased RL-like model of belief updating (pxp = 0.99, Ef = 0.40; Appendix 4, Figure 2—figure supplement 3).

**Figure 3.**
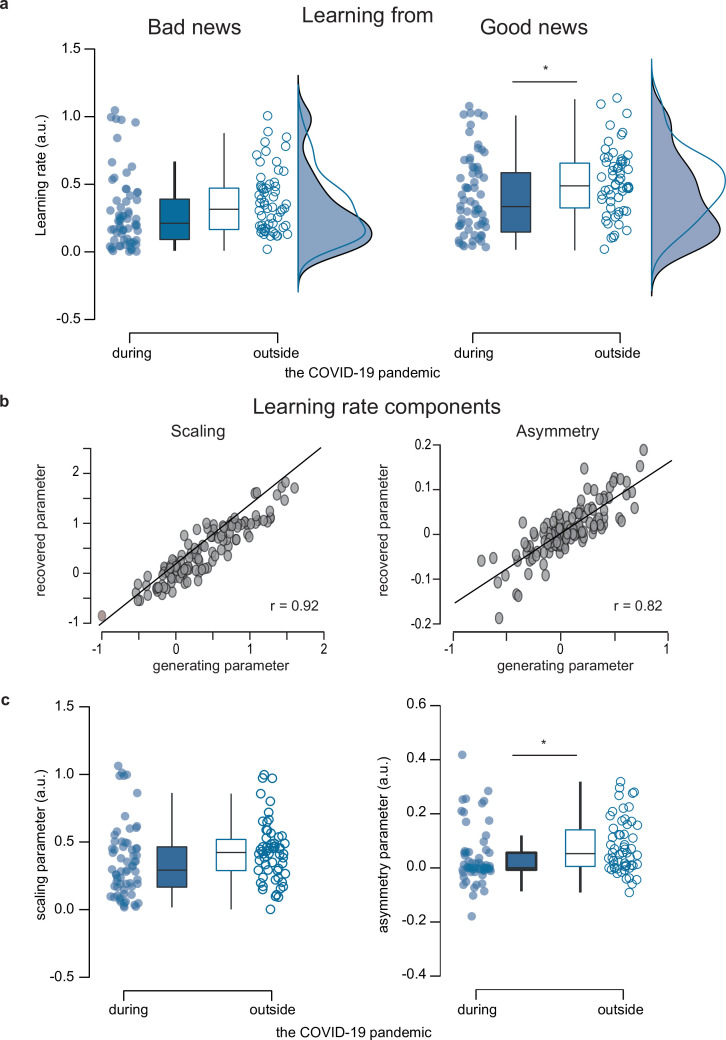
Parameter comparisons between participants tested during (n=65) and outside (n=58) the COVID-19 pandemic. (**a**) Learning rates. Boxplots display 95% confidence intervals for learning rates from the RL-like updating model that assumed updating is proportional to the estimation error with an asymmetry and a scaling learning rate component. (**b**) *Parameter recovery for learning rate components of the overall best fitting Model 1 (n=123)*. Pearson’s correlation between generating and recovered parameters for scaling (left panel) and asymmetry (right panel) learning rate component. r –Pearson’s correlation coefficient against zero. Source data and exact p-values are provided as a Source Data file. (**c**) *Group comparisons for scaling and asymmetry components*. Boxplots display 95% confidence intervals for the learning rate’s scaling (left panel) and the asymmetry (right panel) component. Boxes in all boxplots correspond to the interquartile range from Q1 (25th percentile) to Q3 (75th percentile). The horizontal black lines indicate medians, and whiskers range from minimum to maximum values and span 1.5 times the interquartile range. The dots correspond to individual participants. *p<0.05. p-values were obtained with two-sampled, two-tailed t-tests between groups, and exact values are provided in the source data file. Figure 3—source data 1.Computational model parameters per participant.

A linear mixed effects model (LME), analogous to the LME fitted to observed belief updates, was fitted to the learning rates and detected a main effect of EE valence (ß=0.09, SE = 0.01, t(236) = 7.14, p=1.18e-11, 95% CI [0.06 to 0.11]; Appendix 7—table 9), and a significant interaction EE valence by context (ß=–0.03, SE = 0.02, t(236) = –2.11, p=0.04, 95% CI [-0.07 to –0.002]; Figure 3a, Appendix 7—table 9). A main effect of EE valence (ß=0.08, SE = 0.02, t(105) = 3.22, p=0.002, 95% CI [0.03 to 0.12]; Appendix 7—table 10) and context (ß=–0.10, SE = 0.03, t(105) = –3.10, p=0.003, 95% CI [-0.17 to –0.04]; Appendix 7—table 10) on learning rates was detected when comparing the participants, who were tested both before and during the pandemic. As shown in Figure 3a, all participants' learning rates were lower in response to bad news than to good news. Still, the difference between good and bad news learning rates was significantly reduced for participants tested during the pandemic. In line with the observed belief updating after good and bad news, the effect of context on the learning rates was driven by a decrease in the learning rates from positive estimation errors in participants tested during the pandemic compared to participants tested outside the pandemic (t(121) = 2.17, p=0.03, Cohen’s d=0.39, two-sampled, two-tailed t-test). Both groups did not differ in their learning rates from negative estimation errors (t(121) = 0.87, p=0.39, Cohen’s d=0.16, two-sampled, two-tailed t-test).

**Figure 3—figure supplement 1.**
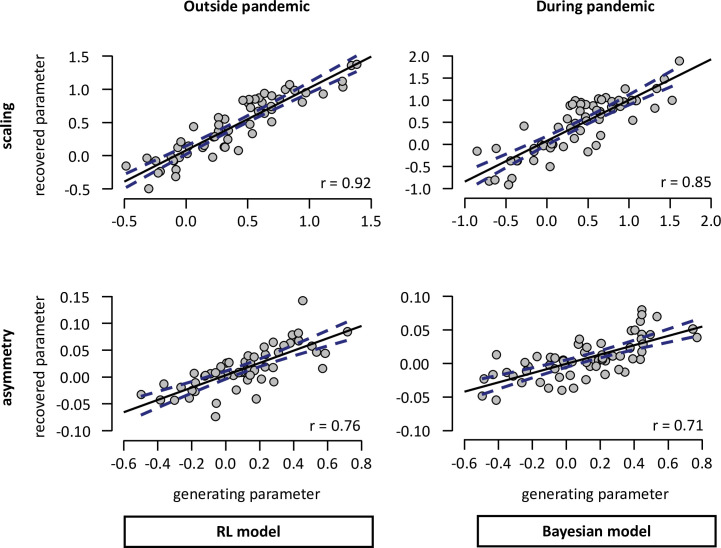
Parameter recovery for the wining model family according to context. Pearson’s correlation between generating and recovered parameters for scaling (upper panel) and asymmetry (lower panel) learning rate component in participants tested outside (n = 58; left panel) and during (n = 65; right panel). The blue doted lines correspond to 95% confidence intervals. r – Pearson’s correlation coefficient against zero. Figure 3—figure supplement 1—source data 1.Pearson correlation coefficients per participant for parameter recovery.

Parameter recovery was successful for the scaling (*r*=0.92, p<0.001) and asymmetry (*r*=0.82, p<0.001) components of the learning rates (Figure 3b), which indicated that the model gave identifiable values for these parameters (parameter recovery was also conducted on each group and each model family separately, results are reported in Appendix 4 and Figure 3—figure supplement 1). We, therefore, were able to explore potential group differences in the learning rate components in more detail. Linear mixed effects modeling found a main effect of context for the asymmetry component (ß=–0.04, SE = 0.02, t(117) = –2.32, p=0.02, 95% CI [-0.07 to –0.01]; Figure 3c, Appendix 7—table 11), but not for the scaling component (ß=–0.07, SE = 0.05, t(117) = –1.54, p=0.13, 95% CI [-0.16 to 0.02]; Figure 3c, Appendix 7—table 12). The average asymmetry of learning rates was positive in both groups but significantly smaller in participants tested during the pandemic than those tested outside (t(121) = 2.00, p=0.048, Cohen’s d=0.36, two-sampled, two-tailed t-test, Figure 3c). This result indicated that participants considered positive estimation errors more than negative ones but less when experiencing the COVID-19 pandemic. Similar results were found in the within-subject group (n=28), with a significant main effect of context on asymmetry (ß=–0.06, SE = 0.02, t(51) = –3.72, p=0.001, 95% CI [-0.09 to –0.03]; Appendix 7—table 13), but not on scaling (ß=–0.10, SE = 0.05, t(51) = –1.96, p=0.06, 95% CI [-0.21 to 0.003]; Appendix 7—table 14).

## Discussion

This study investigated how experiencing the COVID-19 pandemic impacted the optimism biases in updating beliefs about the future. Belief updating was optimistically biased before the COVID-19 outbreak, faded during the COVID-19 pandemic, and reemerged after the pandemic. The lack of optimistically biased belief updating during the pandemic was related to three effects: (1) a decreased sensitivity to positive belief-disconfirming information, (2) fewer but stronger negative beliefs about the future, and (3) more confidence in base rates. Computational modeling showed that belief updating during the pandemic was best described by a rational Bayesian model. In contrast, an optimistic RL-like model best-approximated belief updating outside the pandemic. Both models showed that the attenuated optimistically biased belief updating during the pandemic was not due to a learning deficit. The groups were similar in how much they integrated overall evidence in favor of or against initial beliefs. On the contrary, it was explained by a diminished learning asymmetry in considering positive belief-disconfirming evidence that paralleled the observed belief-updating behavior.

The finding that optimistically biased belief updating faded during the pandemic favors the hypothesis that experiencing an adverse life event such as a pandemic weakens optimistic outlooks. It further aligns with the body of research that has explored the malleability of the optimistically biased belief updating and information integration under acute threat and stress and mood disorders such as depression (Strunk et al., 2006; Garrett et al., 2014; Korn et al., 2014; Garrett et al., 2018; Kuper-Smith et al., 2020; Czekalla et al., 2021; Bottemanne et al., 2022; Globig et al., 2021). While our findings align with these previous findings, we also observed a difference. Notably, our sample tested during the pandemic considered positive, favorable information less while showing no change in negative, unfavorable information consideration. Differences in populations and task designs might explain these odds. However, it could also be specific to experiencing the COVID-19 pandemic, which involved an immediate, unpredictable, and global health threat with high uncertainty about its outcome and significant psychological repercussions (Rajkumar, 2020; Brooks et al., 2020).

Mental health assessments during the COVID-19 pandemic indicated that anxiety, stress, paranoia, and depression levels were more prevalent in the population (World Health Organization, 2022; Suthaharan et al., 2021). The rapid spread of SARS-CoV-2 and the emergence of COVID-19 cases worldwide constitute a challenging situation that, in 5 months, has shifted from an elusive and distant threat to an immediate and drastic health and economic crisis. All citizens were confronted daily with alarming figures such as infection rates or mortality, and rather mundane everyday activities, from grocery shopping to jogging, became stressful and threatening situations during which one could catch a potentially fatal infection. Moreover, for many, the COVID-19-related lockdowns of social and economic life implied a physical cut-off from friends and relatives, and life plans, routines, and activities were severely disrupted. It also implied a substantial economic risk. Previous research has shown that economic uncertainties, particularly during marked economic inequality and epidemics, can contribute to belief-updating fallacies reflected by the rise of conspiracy theories (Leonard and Philippe, 2021).

We did not collect physiological measures of stress or information about the COVID-19 infection status of participants, which precludes a direct exploration of the immediate effects of experiencing the infection on belief-updating behavior and the potential interaction with anxiety and stress levels. Although subjective ratings of the perceived risk of death from COVID-19 correlated negatively to the beliefs updating bias measured during the pandemic, this result was obtained in a subset of participants, retrospectively (Appendix 5). We thus cannot directly attribute the observed lack of optimistically biased belief updating during the lockdown to psychological causes such as heightened anxiety and stress. This limitation is noteworthy, as the impact of experiencing the pandemic on belief updating about the future could differ between those who directly experienced infection and those who remained uninfected. It is also important to acknowledge that our study was timely and geographically limited to the context of the COVID-19 outbreak in France. Cultural variations and differences in governmental responses to contain the spread of SARS-CoV-2 may have impacted the optimism biases in belief updating differently.

The observed lack of optimistically biased belief updating may be interpreted as an adaptive response to the experience of an unprecedented level of uncertainty and chronic threat during a global crisis. Although we did not have access to anxiety and stress perceptions during and outside the pandemic, our computational modeling results corroborate to some extent this interpretation. Notably, belief updating was more optimistically biased RL-like outside the pandemic and more rational Bayesian-like during the pandemic. The biased RL-like updating behavior observed outside the pandemic indicated that participants relied on the motivational salience of positive estimation errors to teach them how to update their beliefs about the future by trial and error. This finding aligns with past work, showing that RL-like updating models best explain belief updating in non-threatening, non-stressful, and predictable laboratory contexts (Kuzmanovic and Rigoux, 2017). It further suggests that RL strategies are a computationally efficient way to guide decision-making and belief formation when the environment is stable and predictable (Gershman and Daw, 2017). For instance, in environments with well-defined reward structures, the human brain has been shown to efficiently rely on RL and avoid a computational overhead associated with the Bayesian-like inference process (Daw et al., 2005). On the contrary, belief updating was more rational Bayesian-like during the COVID-19 pandemic, indicating that participants weighed the uncertainty of evidence in favor of and against their prior beliefs. This finding aligns with research about Bayesian networks to model semantic knowledge processing under uncertainty (Pearl, 1988) and with work that uses Bayes rule to understand how humans learn and choose under uncertainty (Tenenbaum et al., 2011; Griffiths and Tenenbaum, 2006; Gershman et al., 2015).

It is essential to acknowledge that computational modeling provides insight into potential mechanisms, but this excludes inferences on whether humans indeed update beliefs in the way the best-fitting model assumes. Other models, such as evidence accumulation models, may also work when humans update their beliefs about the future and their immediate surroundings (Gesiarz et al., 2019). Unfortunately, we did not assess reaction times during belief updating, which is crucial for fitting evidence accumulation models such as drift-diffusion models to observed behavior. However, we can infer from our findings that the two model families employed to fit observed belief-updating behavior represented two different but complementary prediction strategies. These strategies were then used to function in the uncertainty of real-life conditions. We call for more studies investigating these computational models' psychological and biological validity under certainty and uncertainty.

In this study, we tested how actual adverse experiences affect the updating of negative future outlooks in healthy participants and in analogy to studies conducted in depressed patients (Garrett et al., 2014; Korn et al., 2014; Bottemanne et al., 2022) following the cognitive model of depression (Beck et al., 1979). One open question is whether findings were specific to the adverse event framing (Harris and Hahn, 2011; Shah et al., 2016; Burton et al., 2022). We argue that under normal, non-adverse contexts, belief updating should also be optimistically biased for positive life events, as shown by previous research (Marks and Baines, 2017; Garrett and Sharot, 2017). However, how context such as experiencing a challenging or favorable situation influences the updating of beliefs about positive and negative outlooks remains an open question.

In conclusion, our results provide insight into the resilience and adaptability of belief-updating processes during and following the COVID-19 pandemic. They demonstrate the malleability of the human ability to anticipate the future and how it can adapt to real-life conditions under which an overly optimistic view of future risks would be harmful.

## Methods

### Ethical considerations

The Local Ethics Committee of Sorbonne University approved the study. All participants provided informed consent and consent to publish. The study protocol followed the Declaration of Helsinki. The authors declare no competing interests.

### Participants

One hundred twenty-five participants (mean age = 37.50 ± 1.28, 99 females) allotted to four different groups were recruited for the study (see Table 1; Appendix 7—table 15) via a public advertisement. Two participants from the group tested in June 2022 were excluded from the analyses because they always indicated the same risk estimate for each event.

**Table 1.** Sociodemographic data for all four groups (N=123).**♀**: Female; **♂**: Male; Note: education is the number of years completed in higher education after a high school diploma.

	October2019(N=30)	March – April 2020(N=34)	May2021(N=31)	June2022(N=28)
Age (years)	34±2	42±3	42±3	35±3
Gender	18 **♀**, 12 **♂**	25 **♀**, 9 **♂**	20 **♀**, 11 **♂**	14 **♀**, 14 **♂**
Education (years)	5±0.4	4±0.3	5±0.2	4±0.4

### Experimental design

**Figure 4.**
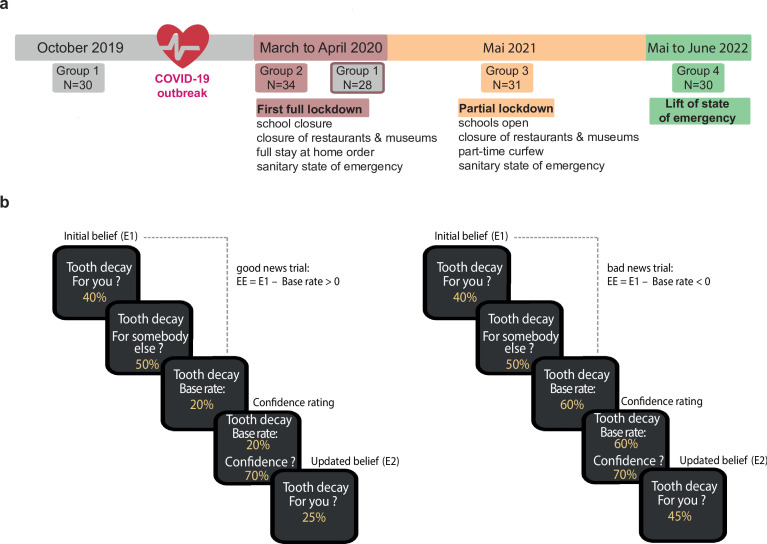
Experimental design. (**a**) Timeline of testing. Four groups were tested, before the COVID-19 outbreak in October 2019, during the first complete lockdown of social and economic life in March and April 2020, after a partial lockdown in May 2021, and after the lift of the pandemic-related state of emergency in June 2022. (**b**) *Belief updating task*. Panels show subsequent appearances on the screen within a good news trial (left panels) and a bad news trial (right panel). Responses were self-paced. The task goal was to estimate the risk of experiencing different adverse future life events (e.g. tooth decay) for oneself (E1) and for somebody else (eBR) before and after (E2) being presented with information about the event’s prevalence in the general population (i.e. base rate (BR)).

The first group of 30 participants (mean age = 33.73 ± 1.96, 18 females) was recruited in October 2019 before the COVID-19 outbreak in France (Figure 4a). These participants were tested in the laboratory. A second group of 34 participants (mean age = 42.24 ± 3.34, 25 females) was recruited from March to April 2020 for online testing during the first COVID-19-related lockdown of social and economic life, with schools closed. A third group of 31 participants (mean age = 42.42 ± 3.35, 20 females) was recruited and tested online immediately after the last lockdown and still during the COVID-19 pandemic in May 2021. A fourth group of 30 participants (mean age = 34.66 ± 2.71, 16 females) was recruited at the lift of the COVID-19 pandemic-related state of emergency and tested in the laboratory in June 2022 (Figure 4a). This group was also used to rule out an eventual effect of task design. Half of them (n=15) performed a one-run task design, and the other half (n=15) performed a two-run task design (e.g. see in more detail the belief updating task description below). Note the 30 participants tested before the COVID-19 pandemic were recontacted during the first strict lockdown to re-perform the belief updating task online (Figure 4a). This allowed us to check for the effects of experiencing a COVID-19-related lockdown within the same cohort of participants. Two of the 30 participants in this group did not respond. Therefore, the sample size for the within-group test-retest analyses was 28 participants.

### Sample sizes

The sample sizes were determined by a power analysis using the *power curve* function in R (version 1.2.5033) and building on the good news/bad news bias observed in the first group tested in October 2019 before the COVID-19 outbreak in France. The sample size required to replicate a significant effect of estimation error valence on the updating with a power between 80% and 90% lay between 28 and 35 participants, respectively.

### Belief updating task

All participants performed a belief-updating task (Figure 4b). For in-person testing, stimulus presentation and response recording were done with the Psychophysics toolbox in MATLAB (R2018b, Update 6, version 9.5.0.1265761). The online testing was done using Qualtrics (Qualtrics Software, version March 2020 of Qualtrics, Copyright 2020 Qualtrics. Available at https://www.qualtrics.com).

The task comprised 40 trials with 40 adverse lifetime events and base rates. In each trial, participants were asked to estimate the likelihood of experiencing an adverse event in the future for themselves and somebody else before and after receiving information about the likelihood of occurrence in the general population (i.e. the base rate). The adverse life events and their actual base rates were taken from previously published work in healthy controls (Sharot et al., 2011; Sharot and Garrett, 2022). The base rates for events were normal to uniformly distributed (W=0.952, p=0.088, Shapiro-Wilk test). The base rates ranged between 10% and 70%, with a mean of 40%. Participants rated their estimates between 3% and 77%, which ensured that for most likely (base rate = 70%) and most unlikely events (base rate = 10%) there was enough space (7%) to update beliefs toward the base rates (Garrett and Sharot, 2017; Sharot and Garrett, 2022). Moreover, all statistical models included the absolute estimation errors as a control for variance potentially explained by different estimation error magnitudes (Garrett and Sharot, 2017; Sharot and Garrett, 2022).

In more detail, as illustrated in Figure 4b, each of the 40 trials began with presenting an adverse life event. Participants estimated their own risk and subsequently the risk of someone else their age and gender. Then the base rate of the event occurring in the general population was displayed on the computer screen. Participants rated their confidence in the accuracy of the presented base rate. Finally, they re-estimated their risk of experiencing the event now informed by the base rate.

The task design varied between some groups:

Fifty-eight participants underwent assessment outside the COVID-19 pandemic, with 45 performing a two-run task design (n=30 tested before the outbreak in October 2019; n=15 tested at the end of the sanitary state of emergency in June 2022). The remaining 13 participants tested outside the pandemic performed the one-run task design like the 65 participants tested during the pandemic.

In the two-run task design, participants performed a first run of 40 trials. Each trial started with the display of an adverse lifetime event. Participants were asked to estimate the risk of experiencing this event in the future for themselves (E1 rating) and for somebody else (eBR rating). At the end of each trial, they received information about the event’s base rates and rated their confidence. In a second run, they saw an adverse future life event and its base rate on each trial. They then re-estimated their risk (E2 rating) on a trial-by-trial basis.

The one-run task design is displayed in Figure 4b and consists of one run of 40 trials. Within each trial, participants first estimated the risk of experiencing a future adverse lifetime event for themselves (E1 rating) and for somebody else (eBR rating), were presented with the base rate for this event (BR), rated their confidence in the base rate and re-estimated their risk of experiencing the event in the future (E2 rating). Note that all analyses were controlled for these differences in task design, which had non-significant effects on belief updating, confidence ratings, estimation error magnitude, and learning rates (see corresponding tables in Appendix 7 with LME results).

### Belief updating task measures of interest

The estimation error indicated whether participants overestimated or underestimated their likelihood of experiencing an adverse event (E1) relative to its actual base rate (BR). The estimation error (EE) was calculated according to the equation i:

(i) $$\displaystyle  \rm EE=E1-BR$$

The estimation error was further used to categorize trials into good or bad news trials:

For good news trials, the estimation error was positive (EE >0), which indicated an overestimation of one’s likelihood of experiencing an adverse life event relative to the base rate of that event (E1 >BR). For bad news trials, the estimation error was negative (EE <0), which indicated an underestimation of one’s likelihood of experiencing an adverse event relative to its actual base rate (E1 <BR).

The main variable of interest was the magnitude of belief updating (UPD), which was calculated as the difference between the first (E1) and the second (E2) estimate after receiving information about the base rate (BR). Notably, the update was calculated for good and bad news trials, respectively, following equation ii:

(ii) $$\displaystyle  \begin{array}{ll}if\, EE>0\\\quad \quad \quad {\rm UDP_{good\, news}=E1-E2} \\ if \,EE<0\\\quad \quad \quad {\rm UDP_{bad\, news}=E2-E1}\end{array} $$

Lastly, the difference between updating after good and bad news was calculated to assess the updating bias following equation iii:

(iii) $$\displaystyle  \rm UDB=UPD_{good \,news}-UPD_{bad \,news}$$

A positive difference indicated that participants updated their beliefs about their lifetime risk of experiencing adverse life events more frequently following good news than bad news.

For each participant, trials that did not receive a response (on average 0.44 trials per subject) and trials with an EE = 0 (on average 0.63 trials per subject) were excluded from the analyses.

The distance measured the extent to which participants consider their probability of experiencing a given adverse event (E1) different from the lifetime risk of someone from a similar socio-economic background (eBR). If positive, it reflected an optimistic bias in initial estimates. The following, distance = eBR – E1, was calculated. Additional analysis to control for this measure was added (Appendix 7—table 3).

### Model-free statistical analyses of observed belief updating behavior

The main aim of this study was to assess how belief updating was affected by the context of experiencing the COVID-19 pandemic.

We, therefore, conducted between-context analyses, contrasting groups tested during (i.e. during the first lockdown in March/April 2020 and immediately after the last lockdown in May 2021) and outside the pandemic context (i.e. before the outbreak in October 2019 and 1 year after the last lockdown in June 2022). All statistical tests were conducted using the MATLAB Statistical Toolbox (MATLAB 2018b, MathWorks) and JASP (JASP 0.16.4).

A first linear mixed effects model (LME 1) was fitted to the belief updating, following equation *iv*:

(iv) $$\displaystyle  \begin{array}{ll}\rm UPD=\beta_{0}\,Intercept \,+ \beta_{EE}\,|EE| +\beta_{EE} valence + \beta_{context}\,context+\beta_{design} \\ \rm design+\beta_{age}\,age +\beta_{gender}\,gender+\beta_{education}education\, +\rm \beta_{EE \,valence\,*\,context}\\\rm \,EE \,valence \, by\, context +(1\,|\,subject)+(1+\,|EE|\,|subject)+(1+EE \, valence \, | \ \\ \rm subject)\end{array}$$

The model included fixed effects for estimation error magnitude (|EE|), estimation error valence (EE valence, coded –1 for bad news trials and 1 for good news trials), context (coded 0 for outside and 1 for during the COVID-19 pandemic), task design (coded 1 for one-run, 2 for two-run design), age, gender (coded 0 for male, 1 for female participants), level of education, and the interaction of interest EE valence by context. The model also included random intercepts nested by subject number and random slopes for estimation error magnitude and valence.

Subsequently, the same Linear Mixed Effects (LME) model was applied again to the belief update to explore the categorical effect of context in conjunction with EE valence. This allowed for a more specific comparison of the impact of EE valence between contexts (groups): those tested before the COVID-19 pandemic outbreak in October 2019 (baseline) compared to those tested during the initial COVID-19-related lockdown (context 1), those tested immediately after the last lockdown during the pandemic in May 2021 (context 2), and those tested one year post-pandemic in June 2022 (context 3), respectively.

Post-hoc two-tailed and one-tailed t-tests were conducted to characterize the directionality of detected main effects and interactions.

### Posthoc power analysis

The best fitting computational models of belief updating in each context (i.e. during and outside the pandemic) and their free parameters were used to simulate new belief updates (Wilson and Collins, 2019). Simulations were repeated 1000 times. At each iteration, the above-described linear mixed effects model (equation iv) was fitted to the simulated belief updates. The frequency across 1000 iterations with which the LME detected a significant interaction of valence by context on simulated belief updating indicates the power of this interaction effect and the chance for type II errors of failing to reject the null hypothesis when the effect was there.

### Model-based analyses of belief-updating behavior

To gain more insight into putative cognitive mechanisms of belief updating during and outside the COVID-19 pandemic, two families of non-linear computational models were fitted to observed belief updating behavior, which is specified below.

### Model specifications

#### Reinforcement learning model of belief updating

A Reinforcement learning-like model assumed that belief updating is proportional to the magnitude of the estimation error following Kuzmanovic and Rigoux, 2017. The learning rate scaled the effect of the estimation error on belief updating following the generic equation v:

(v) $$\displaystyle  \rm UPD=LR*EE$$

Importantly, the learning rate was estimated for good and bad news trials separately and following equations *vi* and *vii*:

(vi) $$\displaystyle  \rm LR_{good \, news}=S+A$$

(vii) $$\displaystyle  \rm LR_{bad \, news}=S-A$$

For both types of trials, the learning rate was composed of two components that varied across participants. The scaling parameter (S) measured the extent to which a participant took the estimation error into account when updating beliefs. The asymmetry parameter (A) indicated to what extent the belief updating differed for positive and negative estimation errors. The priors for scaling and asymmetry were untransformed and unbound. The mean of the prior distribution for scaling was set to one. Thus, a scaling of one meant that the updating magnitude equaled the estimation error magnitude. The mean of the prior distribution for the asymmetry parameter was set to zero. An asymmetry parameter value larger than zero meant positively biased updating, whereas an asymmetry parameter smaller than zero meant negatively biased belief updating.

A version of the RL-like model of belief updating took the personal relevance (PR) of presented adverse future life events into account following equations viii and ix:

(viii) $$\displaystyle  \rm UPD_{good\, news}=(S+A)* EE*(1-PR)$$

(ix) $$\displaystyle  \rm UPD_{bad\, news}=(S-A)*EE*(1-PR)$$

The PR weighed the estimation error (EE) and corresponded to the difference between the estimated base rate (eBR, the estimated risk for somebody else) and the initial estimate (E1, the estimated risk for oneself). Based on the sign of this difference between eBR and E1, the PR was calculated following equations x to xii:

(x) $$\displaystyle  \begin{array}{ll}\, If \, eBr<E1\\ \quad \quad \quad PR=\frac{eBR- E1}{eBR- 1}\end{array}$$

(xi) $$\displaystyle  \begin{array}{ll}\, If\, eBR>E1 \\ \quad \quad \quad PR=\frac{E1- eBR}{99- eBR}\end {array}$$

(xii) $$\displaystyle  \begin{array}{ll}\, If\, eBR=E1\\ \quad \quad PR=0\end{array}$$

#### Bayesian belief updating model

A second family of computational models was fitted to belief updating behavior and assumed that belief updating was proportional or equal to the Bayes rule, following equations xiii and xiv (Kuzmanovic and Rigoux, 2017):

(xiii) $$\displaystyle  \rm UPD_{good\, news}=(E1-E2b)*(S+A)$$

(xiv) $$\displaystyle  \rm UPD_{bad\, news}=(E2b-E1)*(S-A)$$

The scaling parameter (S) corresponded to the tendency of participants to update their beliefs in response to the presented base rate following Bayes' rule. A scaling smaller than one (S<1) indicated lesser belief updating than what was predicted by the Bayes rule, and a scaling larger than one (S>1) indicated more updating than predicted by the Bayes rule.

The Bayes rule was used to define a Bayesian second estimate (E2b, the updated belief), which was calculated following equations xv and xvi:

(xv) $$\displaystyle  E2b=\rm Prior*LHR$$

(xvi) $$\displaystyle  E2b=\rm \frac{Prior*LHR}{1+(Prior*LHR)}$$

With the Prior=P(BR), corresponding to the base rate (BR) of each event following equation xvii:

(xvii) $$\displaystyle  Prior=\rm \frac{BR}{1-BR}$$

The Likelihood Ratio (LHR) indicates the probability of the initial estimate (E1) relative to the likelihood of the alternative estimated base rate (eBR) following equation xviii:

(xviii) $$\displaystyle  LHR=\rm \frac{\frac{E1}{1-E1}}{\frac{eBR}{1-eBR}}$$

Alternative models of these two model families (RL and Bayesian) were fitted to the observed belief-updating behavior. Each model alternative represented a different combination of free parameters composing the learning rate to test a total of 12 assumptions about the cognitive process underlying belief updating:

RL model 1. Belief updating is asymmetrical and proportional to the estimation error: S+A varied across participants.
RL model 2. Belief updating is non-asymmetrical and proportional to the estimation error: S varied, A was silent (fixed to zero).
RL model 3. Asymmetrical updating is equal to the estimation error: S was fixed (to one), and A varied.
RL model 4. Updating equals the estimation error: S and A were fixed.
RL model 5. Belief updating is asymmetrical, proportional to the estimation error, and moderated by the personal relevance of events (PR): S+A varied. PR was weighting the EE following equations x., xi., and xii.
RL model 6. Belief updating is non-asymmetrical and proportional to the estimation error moderated by PR: S varied, A was fixed, and PR weighted the EE following equations x., xi., and xii.
RL model 7. Asymmetrical updating equals the estimation error moderated by PR: S was fixed, A varied, and PR weighted the EE following equations x., xi., and xii.
RL model 8. Updating equals the estimation error moderated by PR: S and A were fixed, and PR weighted the EE following equations x., xi., and xii.
Bayesian model 1. Belief updating is asymmetrical and proportional to Bayes rule: S and A varied.
Bayesian model 2. Belief updating is proportional to a rational Bayes rule: S varied, and A was fixed.
Bayesian model 3. Belief updating equals an asymmetrical Bayes rule: S was fixed, and A varied.
Bayesian model 4. Belief updating equals a rational Bayes rule: S and A were fixed.

### Model estimation

Models were estimated following the procedure reported by Kuzmanovic and Rigoux, 2017 and Bottemanne et al., 2022. In short, models were not hierarchical, and parameter estimation was thus less sensitive to differences in group sample sizes. For each participant, optimal scaling and asymmetry parameter values were obtained using Bayesian variational inferences implemented in the VBA toolbox (Daunizeau et al., 2014).

### Model comparisons

The free energy approximations for a model’s evidence in each participant were entered into a random effect Bayesian model comparison that yielded the two criteria considered for model selection: the estimated model frequency (Ef) in each group (estimate the frequency of each model in the population) and the protected exceedance probability (pxp), which corresponded to the probability that the hypothesis predominates in the population, above and beyond chance.

### Parameter recovery

Parameter recovery analysis was conducted to check whether the free parameters of the winning models were identifiable and described the data better than any other set of parameters. The procedure was the same as reported in Bottemanne et al., 2022. In short, to validate the accuracy of the fitting procedure in providing meaningful parameter values, simulated belief updating data was generated using the observed parameter values for both the optimistic RL model and the optimistic Bayesian updating model. Subsequently, we applied the fitting procedure to these simulated data to iteratively 'recover' the parameters. Thereby, the means of the parameters were set to correspond to the observed sample means (i.e. scaling = 0.39 ± 0.02, asymmetry = 0.07 ± 0.01 for the RL model; scaling = 0.42 ± 0.03, asymmetry = 0.05 ± 0.01 for the Bayesian model). This process was iterated to simulate 40 values of belief updates 123 times. The model was then inverted by fitting it to the simulated data, yielding a new set of recovered values for scaling and asymmetry. Finally, the recovered and estimated parameters were compared by assessing their correlation using Pearson’s correlation coefficients.

### Parameter comparisons

To compare learning rates and learning rate components across groups, we used the parameters from the optimistically biased RL-like model (RL model 1), which performed best when fitted to the whole dataset (Ef = 0.40, pxp = 0.99) and reproduced the observed updating behavior as shown in Figure 2—figure supplement 3.

Individual learning rates from this RL model 1 and their scaling and asymmetry components were the dependent variables (DV) of the following generic linear mixed effects model (equation xix and xx):

(xix) $$\displaystyle  \begin{array}{ll} LR=\rm \beta_0 Intercept\,+\,\beta_{valence}\, valence\,+\, \beta_{context} \,context\,+\, \beta_{design}\,design\,+\beta_{age}\, age\, +\beta_{gender}\\ \rm gender\,+\,\beta_{education}\, education\,+\,\beta_{valence}\, valence\, by \, context +(1|\, subject)\end{array}$$

(xx) $$\displaystyle  \begin{array}{ll}parameters=\rm \beta_{0}Intercept\,+\beta_{context}\,context\,+\beta_{age}\, age\,\beta_{gender}\,gender\,\beta_{education}\\ \rm education\,+(1|subject)\end{array}$$

The model included fixed effects for news valence (valence, coded 1 for good news, –1 for bad news), context (coded 0 for outside the pandemic, 1 for during the pandemic), task design (coded 1 for one-run and 2 for two-run), age, gender (coded 0 for male, 1 for female participants), and level of education. It also tested the interaction of interest context by valence. The intercept was nested at random by subject number.

Post-hoc one-sampled and two-sampled t-tests were conducted to characterize the directionality of effects.

### Model recovery

To check if the 12 models were identifiable, a model recovery analysis was conducted using the VBA toolbox (Daunizeau et al., 2014). In more detail, behavior was simulated using each of the 12 models with parameters estimated from participants' actual behavior. These simulated datasets were then refitted to all alternative models. The model comparison procedure was then performed to evaluate whether each model could accurately recover the parameters that generated the data. This resulted in a 12x12 confusion matrix that compared the performance of all models in fitting each simulated dataset (Figure 2—figure supplement 2). The matrix shows the estimated frequency when fitting to the 12 models (y axis), the behavior generated by each model (x axis), and provided evidence for strong recovery of nearly all models and, importantly, the two winning models: the optimistically biased RL-like model and the rational Bayesian model of belief updating. This analysis thus rules out that the two model families were confused and mitigates concerns about the validity of the model selection.

## Data Availability

Source data files to generate the figures and figure supplements have been provided.

## Additional files

MDAR checklist

## Appendix 1

### Within-group comparisons

Belief updating was compared within a group of participants (n=28), who were tested both before (October 2019) and during the COVID-19 pandemic (March-April 2020). A mixed effects linear regression analysis of belief updating showed a significant main effect of EE valence (*β*=4.05, SE = 1.22, t(103) = 3.31, p=0.001, 95% CI [1.63–6.47]), as well as a significant effect of testing context (*β*=–4.41, SE = 1.49, t(103) = –2.95, p=0.004, 95% CI [-7.37––1.45]), and more importantly a significant EE valence by testing context interaction (*β*=–7.66, SE = 1.49, t(103) = –5.13, p<0.001, 95% CI [-10.62 to –4.70], Figure 1—figure supplement 1, Appendix 7—table 4). A significant main effect of gender was also found (*β*=3.53, SE = 1.64, t(103) = 2.15, p=0.03, 95% CI [0.27–6.79]), with women updating their beliefs more than men. Post-hoc t-tests revealed that participants tested before the emergence of the pandemic updated their beliefs more after receiving good news (mean UPD_good_ = 14.38 ± 1.14) than after receiving bad news (mean UPD_bad_ = 6.23 ± 1.17; t(27) = 4.93, p<0.001, Cohen’s d=0.93, paired sample, two-tailed t-test). This asymmetry in belief updating was not observed when the same 28 participants were tested during the first lockdown (mean UPD_good_ = 2.25 ± 1.76; mean UPD_bad_ = 9.30 ± 2.47; t(27) = –1.84, p=0.08, Cohen’s d=–0.35, paired sample, two-tailed t-test).

Bayesian model comparison in the group of participants tested both before and during the lockdown showed that belief updating was more RL-like and optimistic before the lockdown (Ef = 0.73, pxp = 1), and rational Bayesian-like during the lockdown (Ef = 0.61, pxp = 0.99; Figure 2—figure supplement 1).

## Appendix 2

### Optimism bias in initial beliefs

As shown in Figure 1—figure supplement 2, the participants displayed an optimism bias in their initial belief estimates: They estimated that adverse events are more likely to happen to others than to themselves (*β*=3.02, SE = 0.86, t (232)=3.53, p=0.001, 95% CI [1.33–4.71]; Appendix 7—table 18). However, the pandemic context had no significant effect (*β*=–1.91, SE = 3.00, t (232)=–0.64, p=0.52, 95% CI [-7.82–4.00]; Appendix 7—table 18). We conclude from these additional analyses that the pandemic context specifically influenced optimistically biased belief updating but did not affect optimism bias in initial beliefs.

Note that optimism bias is the propensity to believe that negative events are more likely to happen to others than to oneself. In contrast, the terms optimistically biased belief updating or optimism biases in belief updating used throughout the main text refer specifically to the valence effect in belief updating —updating beliefs more in response to good news than to bad news.

## Appendix 3

### Sources of variance in belief updating

In Figure 1b, participants tested during the COVID-19 pandemic showed more variance in belief updating in response to both good and bad news than those tested outside the pandemic. This variability might be because they ignored the base rates when updating their beliefs, possibly influenced by the shift to online testing during the pandemic.

If participants paid attention to the base rates, they were expected to update toward the base rate, yielding positive values for the update. On the contrary, ignoring the base rates can be reflected by updating away from the base rate, with second estimates (E2) that, in the case of good news trials, lay above the first estimate (E1; e.g., E1=60%, BR = 40 %, E2=70 %, UPD = E1 - E2 = - 10 %). Likewise, in the case of bad news trials, second estimates may lie below the first estimate (e.g. E1=20 %, BR = 40 %, E2=10 %, UPD = E2 - E1 = –10%). We examined the number of trials with such paradoxical second estimates yielding negative values for the belief update. We found no significant difference (t(121) = 1.77, p=0.08, Cohen’s d=0.32, two-sample two-tailed t-test) between the number of paradoxical trials in participants tested outside (6.09±0.47 trials on average) and during (4.77±0.56 trials on average) the pandemic. This suggests that participants tested online exhibited no greater propensity for paradoxical responses than those tested in person (non-significant effect of context on the number of paradoxical trials: *β*=–0.19, SE = 1.57, t(234) = –0.12, p=0.90, 95% CI [-3.28–2.90]; Appendix 7—table 16).

Second, we tested if the observed variance in belief updating between the groups was due to second estimates that over- and undershot the base rates. For instance, individuals who undershot indicated second estimates below base rates signaling good news (e.g. E1=40 %, BR = 20 %, E2=10 %). In contrast, individuals who overshot indicated second estimates above the base rates signaling bad news (e.g. E1=10%, BR = 20%, E2=40%). Undershooting might indicate an attuned sensitivity to good news and overshooting an attuned sensitivity to bad news. We found that participants tested outside the COVID-19 pandemic undershot more often (on average 4.48±0.46 trials) than they overshot (on average 2.38±0.33 trials; t(57) = 3.76, p<0.001, Cohen’s d=0.49, paired-sample, two-tailed t-test). This propensity aligned with the bias to update beliefs more after good than after bad news in this group. Conversely, participants tested during the pandemic didn't show a significant difference in the number of trials where they under- (2.00±0.32 trials on average) or overshot (3.08±0.73 trials on average; t(64) = –1.34, p=0. 19, Cohen’s d=–0.17, paired-sample, two-tailed t-test), aligning with the absence of the good news/bad news bias in belief updating. Critically, when comparing the two groups, we found a significant positive interaction testing context by type of shooting (*β*=1.66, SE = 0.50, t(233) = 3.33, p=0.001, 95% CI [0.68–2.65]; Appendix 7—table 17). Post-hoc t-tests showed that participants tested outside the COVID-19 pandemic undershot more often than participants tested during the pandemic (t(121) = 4.48, p<0.001, Cohen’s d=0.81, two-sample two-tailed t-test). No differences were observed between the groups regarding overshooting (t(121) = –0.84, p=0.40, Cohen’s d=–0.15, two-sample two-tailed t-test). These findings indicated that participants tested outside the pandemic were more sensitive to good news, corroborating the findings of a more robust good news/bad news bias in belief updating found in this group.

## Appendix 4

### Parameter and model recovery

#### Parameter recovery for the winning model in each group of participants

To ensure that parameter estimates were robust for the best-fitting models within each context (i.e. during and outside the pandemic), we conducted parameter recovery in each group of participants. For the participants tested outside the pandemic scaling and asymmetry parameters were strongly recovered by the RL-like model (Model 1; Figure 3—figure supplement 1, scaling: *r*=0.92, p=0.001; asymmetry: *r*=0.75, p<0.001). For the group tested during the pandemic recovery for the scaling (*r*=0.85, p<0.001) and asymmetry (*r*=0.71, p<0.001) parameters by the Bayesian model of belief updating was good (Figure 3—figure supplement 1).

#### Model recovery and confusion analysis of the computational models of Belief Updating

To ensure the robustness and specificity of our computational models, we performed a model recovery analysis. Using simulated data, we tested whether each of the 12 models in our comparison framework could be correctly identified under the estimation and selection criteria used in our study. Results indicated that all models were well-recovered (Figure 2—figure supplement 2), except for models in both families that use fixed parameters (i.e. scaling = 1 and asymmetry = 0), which were better recovered by the model with the scaling parameter fixed but the asymmetry parameter estimated iteratively.

#### Comparison of observed and modeled behavior

To check if the overall best fitting optimistically biased RL model reproduced observed belief updating behavior, we compared the observed belief updates in each participant to the updates predicted by the overall winning model (model 1 – RL model with both parameters estimated). This comparison is depicted in Figure 2—figure supplement 3, which highlights a strong correspondence between the actual data and the modeled estimates.

## Appendix 5

### Correlations between self-reported attitudes during the COVID-19 lockdown and belief updating

Some self-reported anxiety and perceived risk measures experienced during the lockdown were collected in a subset of participants (n=40, counting n=21 tested both before and during the 1^st^ strict lockdown, and n=19 tested solely during the 1^st^ lockdown). These reports were given retrospectively at the time of the release of the 1^st^ lockdown in summer 2020 when the pandemic was still unfolding (Appendix 7—table 1).

Exploratory correlations revealed some noteworthy trends, which though did not survive corrections for multiple comparisons. We found that participants who reported having perceived a bigger risk of death due to contagion were also those who were less optimistically biased when updating their beliefs about adverse future life risks during the first strict COVID-19-related lockdown (*r*=–0.36, p=0.02, Pearson’s correlations).

Moreover, parameter estimates from the computational models of belief updating showed associations with specific survey responses: The Bayesian model’s scaling parameter correlated positively with adherence to distancing measures (*r*=0.41, p=0.01) and negatively with the need for social contact (*r*=–0.37, p=0.02). This result indicated that participants who were updating their beliefs faster were more likely to follow preventive guidelines and felt less social craving. Meanwhile, the asymmetry parameter correlated negatively with mask wearing (*r*=–0.41, p=0.01), positively with physical contact with close others (*r*=0.32, p=0.04) and satisfaction with social interactions (*r*=0.33, p=0.04). This suggests that participants who displayed some asymmetry in belief updating during the COVID-19 pandemic were less likely to comply with mask-wearing rules and more likely to engage in social interactions.

Note that the correlations between these questionnaire measures of behavior during COVID-19 related lockdowns and the free parameters hold for both model families (RL and Bayesian). However, for the sake of parsimony, we reported only correlations to the parameters from the Bayesian model, as it provided the best fit to our observed belief updating behavior during the COVID-19 period.

## Appendix 6

### Belief updating task instructions

The following instructions (translated from French) were displayed to participants prior to performing the task:

This task measures your beliefs about future life events. You will be presented with 40 different future life events, one at a time. At each time you are asked to estimate:

1. The likelihood of the event happening in your future life.
2. The likelihood of the event happening to someone else your age and gender.
  You will then see the base rate for the event and will be asked to:
3. Rate your confidence in the base rate information – how much you believe it to be accurate on a scale between 0 and 100%.
  At the end of each trial and after having seen the base rate for the event, you are again asked to estimate:
4. The likelihood of the event happening in your future life.

Please estimate the likelihoods of the future life events on a scale between a minimum likelihood of 3% and a maximum likelihood of 77%.

## Appendix 7

**Appendix 7—table 1.** Survey responses in n=40 participants tested during the pandemic.

Category	Specific question	Mean	sem
Risk perception	COVID-19 risk	2.9	0.2
	General risk perception	1.8	0.1
	COVID-19 mortality risk	3.1	0.2
Adoption of protective measures	Mask wearing	3.7	0.2
	Social distancing outside home (shops, work)	4.4	0.1
	Hand washing	4.2	0.2
	Social distancing at home	4.0	0.1
	Gloves wearing	1.4	0.1
	Shaking hands, hugging	2.2	0.2
	Leave home for work, errands	3.7	0.1
	Hand sanitizer use	4.0	0.2
Need for social interaction	Social craving	3.8	0.2
	Feeling isolated	2.6	0.2
	Calling friends, parents, family, acquaintances	4.5	0.1
	Losing contact with friends, acquaintances	2.3	0.2
	Social media use	3.6	0.2
	Feeling of isolation from loved ones	2.9	0.2
	Quality of social interactions	3.9	0.1
Mood	Sadness and anxiety	2.5	0.2
Anxiety	Level of anxiety	2.6	0.2
Living	1 – alone, 2 – alone with pet, 3 – couple w/o children, 4 – couple w/ children, 5 – Family	3.3	0.2
Housing	1 – apartment, 2 – apartment w/ outdoor space, 3 – house, 4 – house w/ outdoor space	2.3	0.2
Occupation	1 – no occupation. 2 – remote work. 3 – work	2.2	0.1
Displacement	1 – no displacement, 2 – public transportation, 3 – car	2.4	0.1

5–point Likert scale from 1 to 5, 1 – minimal, 3 – medium, 5 – maximum; sem – standard error of the mean.

**Appendix 7—table 2.** Linear Mixed-Effects Model results fitting the average Belief Updates (UPD) in participants tested outside (n=58) and during (n=65) the pandemic.

UPD ~1 + context +valence + EE+confidence + age+gender + education +design + valence*context + (1 | subject) + (1+valence | subject) + (1+EE | subject)												

**Model fit statistics:**												
AIC		BIC				LogLikelihood				Deviance		
1851.1		1913.9				–907.54				1815.1		

**Fixed effects coefficients (95% CIs):**												
Name	Estimate		SE		tStat		DF	pValue		95% CIs		
										Lower		Upper
Intercept	–2.1975		5.033		–0.4366		232	0.6628		–12.114		7.7188
valence	3.2418		1.2297		2.6363		232	0.00895		0.81898		5.6646
context	–0.76328		1.9987		–0.3819		232	0.7029		–4.7013		3.1747
EE	0.4187		0.11434		3.662		232	0.00031		0.19343		0.64397
confidence	0.02234		0.03859		0.5789		232	0.56323		–0.0537		0.09836
age	–0.00690		0.03181		–0.2169		232	0.82845		–0.0696		0.05577
gender	–0.62168		1.0909		–0.5698		232	0.56932		–2.7711		1.5277
education	–0.29937		0.29786		–1.005		232	0.31592		–0.8862		0.2875
design	1.797		1.9642		0.9149		232	0.36121		–2.0729		5.6669
valence:context	–5.5395		1.6879		–3.2819		232	0.00119		–8.8652		–2.2139

**Random effects covariance parameters (95% CIs):**												
Group: subject (121 Levels)												
Name1	Name2			Type		Estimate			95% CIs			
									Lower		Upper	
Intercept	Intercept			std		2.0778			NaN		NaN	

Group: subject (121 Levels)												
Name1	Name2			Type		Estimate			95% CIs			
									Lower		Upper	
Intercept	Intercept			std		3.1204			NaN		NaN	
valence	Intercept			corr		–0.38448			NaN		NaN	
valence	valence			std		8.3125			NaN		NaN	
	
Group: subject (121 Levels)												
Name1	Name2			Type		Estimate			95% CIs			
									Lower		Upper	
Intercept	Intercept			std		12.117			6.4527		22.755	
EE	Intercept			corr		–0.99998			NaN		NaN	
EE	EE			std		0.53486			0.31495		0.90834	

Group: Error												
Name		Estimate				95% CIs						
						Lower				Upper		
Res Std		5.0881				NaN				NaN		
												

**Appendix 7—table 3.** Linear Mixed-Effects Model results fitting the average Belief Updates (UPD) in participants tested outside (n=58) and during (n=65) the pandemic, corrected for distance defined by the difference between the estimate for oneself (E1) and for others (eBR).

UPD ~1 + context +valence + EE+confidence + distance +age + gender +education + design +valence*context + (1 | subject) + (1+valence | subject) + (1+EE | subject)															
	
**Model fit statistics:**															
AIC		BIC					LogLikelihood				Deviance				
1852.8		1919.1					–907.42				1814.8				
	
**Fixed effects coefficients (95% CIs):**															
Name	Estimate			SE		tStat		DF	pValue		95% CIs				
											Lower			Upper	
Intercept	–1.6107			5.1877		–0.3105		231	0.7565		–11.832			8.6105	
valence	3.2539			1.2307		2.6439		231	0.0088		0.82908			5.6788	
context	–0.7479			1.9932		–0.3752		231	0.7078		–4.6751			3.1793	
EE	0.4324			0.11745		3.6817		231	0.0003		0.201			0.66382	
confidence	0.0191			0.0391		0.4892		231	0.6252		–0.0578			0.0961	
distance	–0.0619			0.1262		–0.500		231	0.6239		–0.3105			0.1866	
age	–0.0075			0.0317		–0.2371		231	0.8128		–0.0700			0.0550	
gender	–0.6504			1.0889		–0.5973		231	0.5509		–2.7959			1.4951	
education	–0.3289			0.3053		–1.0775		231	0.2824		–0.9304			0.2725	
design	1.8887			1.9654		0.9610		231	0.3376		–1.9837			5.7611	
valence:context	–5.5767			1.6903		–3.2992		231	0.0011		–8.9071			–2.2463	
	
**Random effects covariance parameters (95% CIs):**															
Group: subject (121 Levels)															
Name1	Name2				Type			Estimate		95% CIs					
										Lower			Upper		
Intercept	Intercept				std			3.3422		NaN			NaN		
valence	Intercept				corr			–0.3646		NaN			NaN		
valence	valence				std			8.3923		NaN			NaN		
	
Group: subject (121 Levels)															
Name1	Name2				Type			Estimate		95% CIs					
										Lower			Upper		
Intercept	Intercept				std			12.344		NaN			NaN		
EE	Intercept				corr			–0.9994		NaN			NaN		
EE	EE				std			0.5443		0.3238			0.9149		

Group: Error															
Name			Estimate					95% CIs							
								Lower				Upper			
Res Std			4.8289					NaN				NaN			
															

**Appendix 7—table 4.** Linear Mixed-Effects Model results fitting the average Belief Updates (UPD) in participants tested before the COVID-19 outbreak in France (October 2019, n=30, baseline), and comparing them to participants tested during the first lockdown in March/April 2020 (n=34, context 1), 1 year later in May 2021 with less strict measures in place (n=31, context 2), and at the lift of the sanitary state of emergency in June 2022 (n=28, context 3).

UPD ~1 + context +valence + EE+confidence + age+gender + education +design + valence*context + (1 | subject) + (1+valence | subject) + (1+EE | subject)												

**Model fit statistics:**												
AIC		BIC				LogLikelihood				Deviance		
1857		1933.8				–906.5				1813		

**Fixed effects coefficients (95% CIs):**												
Name	Estimate		SE		tStat		DF	pValue		95% CIs		
										Lower		Upper
Intercept	–5.041		6.1791		–0.8158		228	0.41546		–17.216		7.1345
valence	4.2371		1.6732		2.5324		228	0.012		0.94033		7.534
EE	0.42432		0.11572		3.6668		228	0.00031		0.1963		0.65233
confidence	0.02225		0.0386		0.57687		228	0.5646		–0.0538		0.098252
age	–0.0062		0.0317		–0.1955		228	0.8452		–0.0686		0.0562
gender	–0.4786		1.1083		–0.4319		228	0.6663		–2.6625		1.7052
education	–0.2686		0.2992		–0.8978		228	0.3703		–0.8582		0.3210
design	2.7278		2.2643		1.2047		228	0.2296		–1.734		7.1894
Context 1	0.9516		2.6961		0.3529		228	0.7245		–4.361		6.2641
Context 2	0.5282		2.7112		0.1948		228	0.8457		–4.814		5.8703
Context 3	1.7166		1.8127		0.9470		228	0.3446		–1.855		5.2883
Valence by context 1	–7.3853		2.2942		–3.2191		228	0.0015		–11.906		–2.8647
Valence by context 2	–5.5876		2.3627		–2.365		228	0.0189		–10.243		–0.9321
Valence by context 3	–2.1096		2.456		–0.8590		228	0.3913		–6.9489		2.7297

**Random effects covariance parameters (95% CIs):**												
Group: subject (121 Levels)												
Name1	Name2			Type		Estimate			95% CIs			
									Lower		Upper	
Intercept	Intercept			std		1.8256			NaN		NaN	

Group: subject (121 Levels)												
Name1	Name2			Type		Estimate			95% CIs			
									Lower		Upper	
Intercept	Intercept			std		2.7322			NaN		NaN	
valence	Intercept			corr		–0.4591			NaN		NaN	
valence	valence			std		8.0804			NaN		NaN	
	
Group: subject (121 Levels)												
Name1	Name2			Type		Estimate			95% CIs			
									Lower		Upper	
Intercept	Intercept			std		12.429			6.8097		22.687	
EE	Intercept			corr		–0.99996			NaN		NaN	
EE	EE			std		0.54549			0.32728		0.90917	

Group: Error												
Name		Estimate				95% CIs						
						Lower				Upper		
Res Std		5.6331				NaN				NaN		
												

**Appendix 7—table 5.** Linear Mixed-Effects Model results fitting the average Belief Updates (UPD) in participants tested both before and during the pandemic (n=28).

UPD ~1 + context +valence + EE+confidence + age+gender + education +valence*context + (1 | subject) + (1+valence | subject) + (1+EE | subject)												

**Model fit statistics:**												
AIC		BIC				LogLikelihood				Deviance		
830.29		876.5				–398.14				796.29		

**Fixed effects coefficients (95% CIs):**												
Name	Estimate		SE		tStat		DF	pValue		95% CIs		
										Lower		Upper
Intercept	10.333		7.5518		1.3683		103	0.1742		–4.6443		25.31
valence	4.0497		1.2219		3.3142		103	0.00127		1.6263		6.4732
context	–4.4101		1.4926		–2.9546		103	0.00388		–7.3704		–1.4499
EE	0.08869		0.14664		0.6046		103	0.54678		–0.2022		0.37948
confidence	0.00141		0.04768		0.02948		103	0.97654		–0.0932		0.09597
age	–0.05015		0.08563		–0.5856		103	0.55941		–0.2199		0.11968
gender	3.5299		1.6424		2.1492		103	0.03396		0.27253		6.7873
education	–0.5649		0.42109		–1.3415		103	0.1827		–1.4		0.27023
valence:context	–7.6601		1.4923		–5.1332		103	1.35e-06		–10.62		–4.7005

**Random effects covariance parameters (95% CIs):**												
Group: subject (28 Levels)												
Name1	Name2			Type		Estimate			95% CIs			
									Lower		Upper	
Intercept	Intercept			std		2.2282e-07			NaN		NaN	

Group: subject (121 Levels)												
Name1	Name2			Type		Estimate			95% CIs			
									Lower		Upper	
Intercept	Intercept			std		1.4291			0.80997		2.5215	
valence	Intercept			corr		-1			NaN		NaN	
valence	valence			std		3.3249			1.9554		5.6537	
	
Group: subject (28 Levels)												
Name1	Name2			Type		Estimate			95% CIs			
									Lower		Upper	
Intercept	Intercept			std		8.274e-07			NaN		NaN	
EE	Intercept			corr		–0.99994			NaN		NaN	
EE	EE			std		3.0154e-08			NaN		NaN	

Group: Error												
Name		Estimate				95% CIs						
						Lower				Upper		
Res Std		7.8366				6.7914				9.0426		
												

**Appendix 7—table 6.** Linear Mixed-Effects Model results fitting the average belief updates (UPD) in participants tested outside (n=58) and during (n=65) the pandemic, corrected for distance, and with estimation errors (EE) calculated based on the estimate for someone else (eBR).

UPD ~1 + context +valence + EE+confidence + distance +age + gender +education + design +valence*context + (1 | subject) + (1+valence | subject) + (1+EE | subject)												

**Model fit statistics:**												
AIC		BIC				LogLikelihood				Deviance		
1822.9		1889.2				–892.44				1784.9		

**Fixed effects coefficients (95% CIs):**												
Name	Estimate		SE		tStat		DF	pValue		95% CIs		
										Lower		Upper
Intercept	–8.2881		5.0873		–1.6292		231	0.1046		–18.311		1.7353
valence	2.4409		1.2913		1.8902		231	0.0600		–0.1034		4.9852
context	–0.7860		1.7408		–0.4515		231	0.6520		–4.2159		2.6438
EE	0.5057		0.1220		4.1459		231	4.8e-05		0.2654		0.7460
confidence	0.0948		0.0349		2.7202		231	0.0070		0.0261		0.1635
distance	–0.1582		0.1054		–1.5013		231	0.1347		–0.3659		0.0494
age	–0.0025		0.0274		–0.0916		231	0.9271		–0.0566		0.0515
gender	0.6055		0.9504		0.6371		231	0.5247		–1.2671		2.4781
education	–0.2419		0.2640		–0.9160		231	0.3606		–0.7621		0.2784
design	0.9142		1.7301		0.5284		231	0.5977		–2.4945		4.3229
valence:context	–5.0968		1.7627		–2.8914		231	0.0042		–8.5698		–1.6237

**Random effects covariance parameters (95% CIs):**												
Group: subject (121 Levels)												
Name1	Name2			Type		Estimate			95% CIs			
									Lower		Upper	
Intercept	Intercept			std		1.5868			NaN		NaN	

Group: subject (121 Levels)												
Name1	Name2			Type		Estimate			95% CIs			
									Lower		Upper	
Intercept	Intercept			std		2.9714			NaN		NaN	
valence	Intercept			corr		0.2994			NaN		NaN	
valence	valence			std		9.2418			NaN		NaN	
	
Group: subject (121 Levels)												
Name1	Name2			Type		Estimate			95% CIs			
									Lower		Upper	
Intercept	Intercept			std		1.1447			NaN		NaN	
EE	Intercept			corr		–0.9956			NaN		NaN	
EE	EE			std		0.1645			NaN		NaN	

Group: Error												
Name		Estimate				95% CIs						
						Lower				Upper		
Res Std		3.9480				NaN				NaN		
												

**Appendix 7—table 7.** Linear Mixed-Effects Model results fitting the average confidence ratings in participants tested outside (n=58) and during (n=65) the pandemic.

confidence ~1 + context +valence + EE+age + gender +education + design +valence*context + (1 | subject)													

**Model fit statistics:**													
AIC		BIC				LogLikelihood				Deviance			
1908.6		1946.9				–943.28				1886.6			

**Fixed effects coefficients**													
Name	Estimate		SE		tStat		DF	pValue			95% CIs		
											Lower		Upper
Intercept	61.051		9.1552		6.6685		233	1.86e-10			43.013		79.088
valence	–0.4795		0.7288		–0.6580		233	0.5112			–1.9154		0.9563
context	14.105		4.524		3.1177		233	0.0021			5.1915		23.018
EE	–0.2398		0.1177		–2.0376		233	0.0427			–0.4717		–0.0079
age	0.0451		0.0747		0.6037		233	0.5466			–0.1021		0.1923
gender	–2.415		2.5148		–0.9603		233	0.3379			–7.3697		2.5397
education	0.0563		0.6898		0.0816		233	0.935			–1.3027		1.4153
design	3.8568		4.6503		0.8294		233	0.4077			–5.3052		13.019
valence:context	0.0969		1.0256		0.0944		233	0.9248			–1.9237		2.1174

**Random effects covariance parameters**													
Group: subject (121 Levels)													
Name1	Name2			Type		Estimate			95% CIs				
									Lower			Upper	
Intercept	Intercept			std		11.892			10.188			13.88	

Group: Error													
Name		Estimate				95% CIs							
						Lower				Upper			
Res Std		7.6935				6.7828				8.7265			
													

**Appendix 7—table 8.** Linear Mixed-Effects Model results fitting the average absolute Estimation Error (EE) in participants tested outside (n=58) and during (n=65) the pandemic.

EE ~1 + context +valence + confidence +age + gender +education + design +valence*context + (1 | subject)													

**Model fit statistics:**													
AIC		BIC				LogLikelihood				Deviance			
1569		1607.3				–773.48				1547			

**Fixed effects coefficients (95% CIs):**													
Name	Estimate		SE		tStat		DF	pValue			95% CIs		
											Lower		Upper
Intercept	24.602		3.5887		6.8553		233	6.32e-11			17.531		31.672
valence	–0.4550		0.4902		–0.9282		233	0.3543			–1.4209		0.5108
context	3.686		1.6893		2.1819		233	0.0301			0.3577		7.0143
confidence	–0.0496		0.0289		–1.7123		233	0.0882			–0.1067		0.0075
age	0.0403		0.0272		1.4801		233	0.1402			–0.0133		0.0939
gender	–1.073		0.9181		–1.1688		233	0.2437			–2.8818		0.7358
education	–0.3296		0.2511		–1.3128		233	0.1906			–0.8243		0.1651
design	1.7037		1.6971		1.0039		233	0.3165			–1.64		5.0473
valence:context	2.1942		0.6688		3.2808		233	0.0012			0.8765		3.5119

**Random effects covariance parameters**													
Group: subject (121 Levels)													
Name1	Name2			Type		Estimate			95% CIs				
									Lower			Upper	
Intercept	Intercept			std		3.046			2.1255			4.3652	

Group: Error													
Name		Estimate				95% CIs							
						Lower				Upper			
Res Std		5.1867				4.5717				5.8845			
													

**Appendix 7—table 9.** Linear Mixed-Effects Model results fitting the average Learning Rates from the RL-like model in participants tested outside (n=58) and during (n=65) the pandemic.

LR ~1 + context +valence + age+gender + education +design + valence*context + (1 | subject)													

**Model fit statistics:**													
AIC		BIC				LogLikelihood				Deviance			
–54.291		–19.319				37.146				–74.291			

**Fixed effects coefficients**													
Name	Estimate		SE		tStat		DF	pValue			95% CIs		
											Lower		Upper
Intercept	0.5189		0.1546		3.3566		236	0.0009			0.2144		0.8235
valence	0.0856		0.0120		7.1351		236	1.18e-11			0.0620		0.1092
context	–0.0492		0.0797		–0.6172		236	0.5377			–0.2062		0.1078
age	–0.0005		0.0014		–0.3404		236	0.7339			–0.0031		0.0022
gender	–0.0287		0.0457		–0.6267		236	0.5315			–0.1187		0.0614
education	–0.0227		0.0126		–1.8091		236	0.0717			–0.0474		0.0020
design	0.0269		0.0822		0.3272		236	0.7438			–0.1351		0.1889
valence:context	–0.0347		0.0164		–2.1126		236	0.0357			–0.0671		–0.0023

**Random effects covariance parameters**													
Group: subject (122 Levels)													
Name1	Name2			Type		Estimate			95% CIs				
									Lower			Upper	
Intercept	Intercept			std		0.22053			0.19016			0.25575	

Group: Error													
Name		Estimate				95% CIs							
						Lower				Upper			
Res Std		0.12808				0.11297				0.1452			
													

**Appendix 7—table 10.** Linear Mixed-Effects Model results fitting the average Learning Rates for RL-like model in participants tested both before and during the pandemic (n=28).

LR ~1 + context +valence + age+gender + education +valence*context + (1 | subject)													

**Model fit statistics:**													
AIC		BIC				LogLikelihood				Deviance			
–15.954		8.5125				16.977				–33.954			

**Fixed effects coefficients**													
Name	Estimate		SE		tStat		DF	pValue			95% CIs		
											Lower		Upper
Intercept	0.3529		0.1869		1.8887		105	0.0617			–0.0176		0.7234
valence	0.0752		0.0234		3.2175		105	0.0017			0.0288		0.1215
context	–0.1024		0.0330		–3.1011		105	0.0025			–0.1679		–0.0369
age	0.0010		0.0035		0.2876		105	0.7742			–0.0060		0.0080
gender	0.1858		0.0704		2.6385		105	0.0096			0.0462		0.3254
education	–0.0216		0.0178		–1.2130		105	0.2279			–0.0570		0.0137
valence:context	–0.0616		0.0330		–1.8662		105	0.0648			–0.1271		0.0039

**Random effects covariance parameters**													
Group: subject (28 Levels)													
Name1	Name2			Type		Estimate			95% CIs				
									Lower			Upper	
Intercept	Intercept			std		0.15167			0.10662			0.21576	

Group: Error													
Name		Estimate				95% CIs							
						Lower				Upper			
Res Std		0.17479				0.15026				0.20332			
													

**Appendix 7—table 11.** Linear Mixed-Effects Model results fitting the average asymmetry in the RL-like model in participants tested outside (n=58) and during (n=65) the pandemic.

asymmetry ~1 + context +age + gender +education + (1 | subject)													

**Model fit statistics:**													
AIC		BIC				LogLikelihood				Deviance			
–204.79		–185.16				109.39				–218.79			

**Fixed effects coefficients**													
Name	Estimate		SE		tStat		DF	pValue			95% CIs		
											Lower		Upper
Intercept	0.0264		0.0323		0.818		117	0.415			–0.0375		0.0903
context	–0.04		0.0172		–2.3202		117	0.0221			–0.0741		–0.0058
age	0.0006		0.0005		1.1752		117	0.2423			–0.0004		0.0016
gender	0.0089		0.0045		2.0024		117	0.0475			0.0001		0.0178
education	–0.0048		0.0172		–0.2815		117	0.7788			–0.0388		0.0291

**Random effects covariance parameters**													
Group: subject (122 Levels)													
Name1	Name2			Type		Estimate			95% CIs				
									Lower			Upper	
Intercept	Intercept			std		0.063415			NaN			NaN	

Group: Error													
Name		Estimate				95% CIs							
						Lower				Upper			
Res Std		0.063415				NaN				NaN			
													

**Appendix 7—table 12.** Linear Mixed-Effects Model results fitting the average scaling in the RL-like model in participants tested outside (n=58) and during (n=65) the pandemic.

scaling ~1 + context +age + gender +education + (1 | subject)													

**Model fit statistics:**													
AIC		BIC				LogLikelihood				Deviance			
10.519		30.147				1.7406				–3.4813			

**Fixed effects coefficients**													
Name	Estimate		SE		tStat		DF	pValue			95% CIs		
											Lower		Upper
Intercept	0.561		0.0858		6.5375		117	1.71e-09			0.3911		0.731
context	–0.0705		0.0458		–1.5406		117	0.1261			–0.1612		0.0201
age	–0.0004		0.0014		–0.3263		117	0.7448			–0.0031		0.0022
gender	–0.0214		0.0119		–1.7993		117	0.0745			–0.0449		0.0022
education	–0.0297		0.0456		–0.6517		117	0.5159			–0.1201		0.0606

**Random effects covariance parameters**													
Group: subject (122 Levels)													
Name1	Name2			Type		Estimate			95% CIs				
									Lower			Upper	
Intercept	Intercept			std		0.16865			NaN			NaN	

Group: Error													
Name		Estimate				95% CIs							
						Lower				Upper			
Res Std		0.16865				NaN				NaN			
													

**Appendix 7—table 13.** Linear Mixed-Effects Model results fitting the average asymmetry in the RL-like model in participants tested both before and during the pandemic (n=28).

asymmetry ~1 + context +age + gender +education + (1 | subject)													

**Model fit statistics:**													
AIC		BIC				LogLikelihood				Deviance			
–138.71		–124.53				76.354				–152.71			

**Fixed effects coefficients**													
Name	Estimate		SE		tStat		DF	pValue			95% CIs		
											Lower		Upper
Intercept	0.0471		0.0473		0.9969		51	0.3235			–0.0478		0.1420
context	–0.0615		0.0165		–3.7185		51	0.0005			–0.0947		–0.0283
age	0.0005		0.0009		0.6121		51	0.5432			–0.0012		0.0023
gender	–0.0023		0.0176		–0.1285		51	0.8983			–0.0376		0.0331
education	0.0019		0.0045		0.4158		51	0.6793			–0.0071		0.0108

**Random effects covariance parameters**													
Group: subject (28 Levels)													
Name1	Name2			Type		Estimate			95% CIs				
									Lower			Upper	
Intercept	Intercept			std		9.1905e-09			NaN			NaN	

Group: Error													
Name		Estimate				95% CIs							
						Lower				Upper			
Res Std		0.06189				0.051427				0.074482			
													

**Appendix 7—table 14.** Linear Mixed-Effects Model results fitting the average scaling in the RL-like model in participants tested both before and during the pandemic (n=28).

scaling ~1 + context +age + gender +education + (1 | subject)													

**Model fit statistics:**													
AIC		BIC				LogLikelihood				Deviance			
3.4809		17.658				5.2595				–10.519			

**Fixed effects coefficients**													
Name	Estimate		SE		tStat		DF	pValue			95% CIs		
											Lower		Upper
Intercept	0.3529		0.1880		1.8776		51	0.0662			–0.0244		0.7302
context	–0.1024		0.0524		–1.9554		51	0.0560			–0.2076		0.0027
age	0.0010		0.0035		0.2876		51	0.7748			–0.0061		0.0081
gender	0.1858		0.0704		2.6385		51	0.0110			0.0444		0.3272
education	–0.0216		0.0178		–1.2130		51	0.2307			–0.0575		0.0142

**Random effects covariance parameters**													
Group: subject (28 Levels)													
Name1	Name2			Type		Estimate			95% CIs				
									Lower			Upper	
Intercept	Intercept			std		0.10692			0.046689			0.24485	

Group: Error													
Name		Estimate				95% CIs							
						Lower				Upper			
Res Std		0.19601				0.15084				0.2547			
													

**Appendix 7—table 15.** Sociodemographical data (N=123).

	N	gender	age	education level
Outside pandemic	58	32 females	33.84±1.68	4.54±0.29
During pandemic	65	45 females	42.32±2.35	4.59±0.19
Group tested before and during	28	18 females	34.14±2.08	5.00±0.41

Note: education is reported as years of higher education (university level).

**Appendix 7—table 16.** Linear Mixed-Effects Model results fitting the average number of paradoxical trials in participants tested outside (n=58) and during (n=65) the pandemic.

Nb of trials ~1 + valence +context + age+gender + education +design + context*valence + (1 | subject)													

**Model fit statistics:**													
AIC		BIC				LogLikelihood				Deviance			
1623.5		1644.6				–805.76				1611.5			

**Fixed effects coefficients**													
Name	Estimate		SE		tStat		DF	pValue			95% CIs		
											Lower		Upper
Intercept	19.427		3.059		6.3508		234	1.10e-09			13.4		25.454
valence	–1.9732		0.6083		–3.2436		234	0.0014			–3.1717		–0.7747
context	–0.1904		1.57		–0.1213		234	0.9036			–3.2836		2.9028
age	0.0010		0.0260		0.0402		234	0.9680			–0.0501		0.0522
gender	–0.1403		0.8746		–0.1604		234	0.8727			–1.8633		1.5828
education	0.0022		0.2397		0.0092		234	0.9926			–0.4701		0.4745
design	0.1215		1.6177		0.0751		234	0.9402			–3.0656		3.3085
context:valence	–2.3576		0.8300		–2.8404		234	0.0049			–3.9928		–0.7223

**Random effects covariance parameters**													
Group: subject (123 Levels)													
Name1	Name2			Type		Estimate			95% CIs				
									Lower			Upper	
Intercept	Intercept			std		2.8591e-15			NaN			NaN	

Group: Error													
Name		Estimate				95% CIs							
						Lower				Upper			
Res Std		6.4381				5.8893				7.038			
													

**Appendix 7—table 17.** Linear Mixed-Effects Model results fitting the average number of under- and overshooting in participants tested outside (n=58) and during (n=65) the pandemic.

Nb of shoot ~1 + type+context + EE+age + gender +education + design +context*type + (1 | subject)													

**Model fit statistics:**													
AIC		BIC				LogLikelihood				Deviance			
1623.5		1644.6				–805.76				1611.5			

**Fixed effects coefficients**													
Name	Estimate		SE		tStat		DF	pValue			95% CIs		
											Lower		Upper
Intercept	3.5229		2.0186		1.7452		233	0.0823			–0.4542		7.5001
type	–1.0751		0.3611		–2.9769		233	0.0032			–1.7866		–0.3636
context	0.2141		0.9390		0.2280		233	0.8199			–1.6359		2.064
EE	–0.0288		0.0406		–0.7102		233	0.4783			–0.1088		0.0511
age	0.0079		0.0155		0.5099		233	0.6106			–0.0226		0.0384
gender	–0.4039		0.5200		–0.7766		233	0.4382			–1.4284		0.6207
education	–0.4182		0.1428		–2.9286		233	0.0037			–0.6995		–0.1369
design	1.4082		0.9612		1.4651		233	0.1443			–0.4855		3.3019
context:type	1.6648		0.5003		3.3277		233	0.0010			0.6792		2.6505

**Random effects covariance parameters**													
Group: subject (123 Levels)													
Name1	Name2			Type		Estimate			95% CIs				
									Lower			Upper	
Intercept	Intercept			std		2.2037e-06			NaN			NaN	

Group: Error													
Name		Estimate				95% CIs							
						Lower				Upper			
Res Std		3.8174				3.492				4.1731			
													

**Appendix 7—table 18.** Linear Mixed-Effects Model results fitting initial beliefs about the likelihood of adverse future life events for oneself (E1) and for others (eBR) in participants tested outside (n=58) and during (n=65) the pandemic. Note the perspective regressor (coded 0 for E1 and 1 for eBR) tested if and how beliefs differed when assessed for oneself than for others.

Estimate ~1 + context +perspective + EE+confidence + age+gender + education +design + context*perspective + (1 | subject)												

**Model fit statistics:**												
AIC		BIC				LogLikelihood				Deviance		
1630.4		1672.3				–803.22				1606.4		

**Fixed effects coefficients (95% CIs):**												
Name	Estimate		SE		tStat		DF	pValue		95% CIs		
										Lower		Upper
Intercept	31.472		6.6809		4.7108		232	4.25e-06		18.309		44.635
perspective	3.0194		0.8564		3.5257		232	0.0005		1.3321		4.7067
context	–1.9127		2.9993		–0.6377		232	0.5243		–7.8221		3.9966
EE	0.3146		0.1261		2.4955		232	0.0133		0.0662		0.5629
confidence	0.0598		0.0350		1.7069		232	0.0892		–0.0092		0.1287
age	–0.0808		0.0481		–1.6793		232	0.09444		–0.1756		0.0140
gender	0.1048		1.6285		0.0643		232	0.9488		–3.1037		3.3133
education	–0.6857		0.4443		–1.5433		232	0.1241		–1.5612		0.1897
design	0.2792		2.9999		0.0931		232	0.9259		–5.6313		6.1897
context: perspective	0.0678		0.9811		0.0691		232	0.9450		–1.8652		2.0007

**Random effects covariance parameters (95% CIs):**												
Group: subject (121 Levels)												
Name1	Name2			Type		Estimate			95% CIs			
									Lower		Upper	
Intercept	Intercept			std		8.0025			6.9443		9.2219	

Group: Error												
Name		Estimate				95% CIs						
						Lower				Upper		
Res Std		3.7506				3.3033				4.2584		
												
